# Hadean bridgmanite in the source of a present-day ocean island

**DOI:** 10.1038/s41586-026-10719-w

**Published:** 2026-07-01

**Authors:** Claudine Israel, Catherine Chauvel, Edward Inglis, Hui Chen, Cécile Hébert, James Badro

**Affiliations:** 1 Université Paris Cité, Institut de Physique du Globe de Paris, CNRS, Paris, France; 2 Electron Spectrometry and Microscopy Laboratory, École Polytechnique Fédérale de Lausanne, Lausanne, Switzerland; 3 Present Address: Department of Earth Sciences, University of Cambridge, Cambridge, UK; 4 Present Address: School of Earth and Environmental Sciences, Cardiff University, Cardiff, UK

**Keywords:** Geochemistry, Geochemistry

## Abstract

Constraining the complex dynamics of the inner Earth unites research efforts across several scientific disciplines, including geochemistry, geophysics and geodynamics. Seismological and geodynamic studies offer insights into the present state of the mantle structure, whereas geochemical approaches characterize its chemical and isotopic heterogeneities^1^, shedding light on the complexity of its evolution. One key challenge is determining the age and origin of its chemical heterogeneities. Here we present new high-precision Nd isotopic measurements in present-day volcanism that identify heterogeneities dating back to the Earth’s earliest history. We report significantly positive ^142^Nd anomalies in lavas from the submarine Fani Maoré volcano in the Comoros archipelago. These anomalies require the preservation, in the mantle, of material depleted in light rare-earth elements (REE) and formed within the first 100 million years (Myr) of Earth’s history. We suggest that this material is mainly composed of bridgmanite that crystallized from an early Earth magma ocean. This Hadean bridgmanite may be more widespread in the present-day mantle than previously expected, raising new questions about its survival over billions of years of plate tectonics and vigorous mantle convection.

## Abstract (web summary)

New high-precision neodymium isotope data measured on recently erupted lavas from the submarine Fani Maoré volcano in the Comoros archipelago allow the identification of heterogeneities dating back to Earth’s earliest history.

## Main

**Fig. 1:**
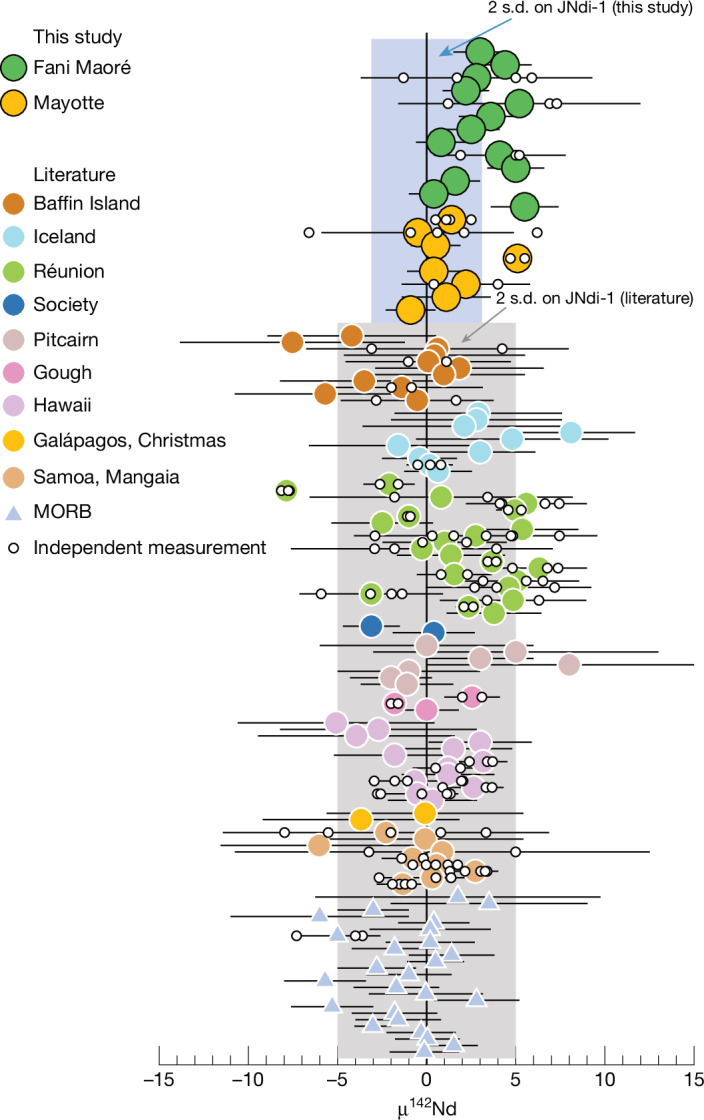
µ^142^Nd anomalies measured in Mayotte and Fani Maoré basalts plotted together with literature OIB and MORB data. µ^142^Nd = ((^142^Nd/^144^Nd_sample_/^142^Nd/^144^Nd_reference_) − 1) × 10^6^ and the reference for the terrestrial composition is JNdi-1 pure solution standard. Error bars correspond to the reproducibility (2 s.d.) when a sample is measured several times and to the internal error (2 s.e.) when measured only once. Blue and grey vertical bands represent the reproducibility on JNdi-1 in this study (3.1 ppm) and typically reported in recent literature (5 ppm), respectively. Data from refs. ^11^–^21^.

Intraplate and ridge volcanism provide valuable and complementary constraints on the structure and composition of the mantle as a whole. Indeed, chemical and isotope variations measured in ocean island basalts (OIB) and in mid-ocean ridge basalts (MORB) demonstrate the inherent presence of heterogeneities formed billions of years ago and preserved despite vigorous convection^1^. Chemical heterogeneities are expected to be increasingly diluted in the mantle over time, making the detection of signatures dating back to the Archaean or Hadean extremely challenging. However, exceptions exist. For instance, anomalous sulfur isotope signatures identified in Mangaia and Pitcairn OIB suggest the preservation in their source of surface material formed before the Great Oxidation Event^2,3^. High ^3^He/^4^He in OIB (for example, Iceland, Samoa, Galápagos, Hawaii) indicates that their source contains primordial mantle material^4^. Also, isotopic heterogeneities of the Hf–W and I–Xe short-lived geochronometers have been reported in OIB, thereby testifying to metal–silicate differentiation events and heterogeneous accretion of volatile-rich materials during the first 50–100 Myr of the Earth’s history^5–7^. Here we focus on the Sm–Nd isotope systems, which provide valuable insights into silicate differentiation, formation of the continents and their subsequent recycling into the mantle. Samarium has two distinct radioactive isotopes, the long-lived ^147^Sm decays into ^143^Nd (half-life of 106 billion years (Gyr) (ref. ^8^)) and the short-lived ^146^Sm decays into ^142^Nd (half-life of 92 Myr (ref. ^9^)). The short half-life of the ^146^Sm–^142^Nd system offers a unique possibility to detect silicate differentiation events that occurred during the first 500 Myr of the Earth. However, the potential variability of the ^142^Nd/^144^Nd ratio is not large. Some Archaean samples preserved in continental settings show excesses and deficits in ^142^Nd up to 20 parts per million (ppm) (see compilation in ref. ^10^), testifying the active decay of ^146^Sm. By contrast, most Phanerozoic rocks have homogeneous ^142^Nd isotope signatures indistinguishable from the modern mantle reference composition. Several studies have sought to identify ^142^Nd isotope variations in modern MORB and OIB (refs. ^11–21^), but most of the published data are within the typical 5-ppm error of the mantle reference composition (see compilation in Fig. 1). To detect differences of less than 5 ppm in ^142^Nd/^144^Nd ratios, greater precision is required. In this study, we present new, high-precision neodymium isotope data measured on recently erupted lavas from Fani Maoré in the Comoros archipelago (details in Methods). Using a new five-line multi-dynamic method for Nd isotopes by thermal ionization mass spectrometry (TIMS) (ref. ^22^), we achieve a 2-year long-term reproducibility of 3.1 ppm (2 s.d.) on the ^142^Nd/^144^Nd ratio of the reference material. Over individual periods of several months, our reproducibility is identical (Methods) and corresponds to the best published errors^23,24^. The reported long-term precision is, to our knowledge, the first of its kind and provides confidence for the identification of remnants of Hadean material in the modern mantle.

We measured the Nd isotope composition of 8 basanites and phonolites from the flank of Mayotte and 13 basanites from Fani Maoré (Fig. 1). The basanites and phonolites from Mayotte have an average µ^142^Nd of +1.3 ±1.3/±3.6 (2 s.e./2 s.d., *n* = 8, with µ^142^Nd = ((^142^Nd/^144^Nd_sample_/^142^Nd/^144^Nd_JNdi-1_) − 1) × 10^6^). By contrast, Fani Maoré basanites have a mean positive µ^142^Nd value of +3.2 ±0.9/±3.3 (2 s.e./2 s.d., *n* = 13). This positive µ^142^Nd value is significantly different from the JNd-1 value, as statistically demonstrated by a *P*-value of 9 × 10^−6^, far below the threshold at 0.05 (see Methods for further details). Both Mayotte and Fani Maoré have indistinguishable ε^143^Nd of 3.93 ±0.11/±0.30 and 3.81 ±0.03/±0.11 (2 s.e./2 s.d.; Methods), respectively. The significant ^142^Nd excess in Fani Maoré lavas indicates that a material formed during the Hadean era and exhibiting a Sm/Nd ratio higher than the bulk silicate Earth (BSE) was preserved in their source for at least 4.3 Gyr.

## Shallow origin of positive ^142^Nd anomalies

A depleted mantle reservoir formed through continental crust extraction in the Hadean era is a recurring candidate^14,25,26^ to explain positive µ^142^Nd values. This process would indeed form a reservoir with a Nd concentration and a Sm/Nd ratio probably similar to those of the present-day depleted MORB mantle (that is, DMM of ref. ^27^). The impact on its ^142^Nd composition depends directly on when the process took place. Continental crust formation and its associated mantle depletion are firmly recorded only after about 3.8 billion years ago (Ga) (ref. ^28^), in which case the mantle µ^142^Nd would be 0. For the sake of the argument, if we assume a crustal extraction age at 4.3 Ga (refs. ^10,29^), we end up with a present-day µ^142^Nd of about +6 for that mantle source. Matching the Fani Maoré µ^142^Nd and ε^143^Nd values requires the plume to consist of approximately 90% of this depleted material (see Methods for details), a volume so substantial that it raises serious questions about its preservation^30^ within the convecting mantle for about 4.3 Gyr. Ideally, we need a process that generates a reservoir that is either large enough so that a small fraction of it can realistically be preserved and/or a reservoir that has a much more positive ^142^Nd anomaly so that smaller quantities would produce the observed effects.

## Magma ocean solidification

**Fig. 2:**
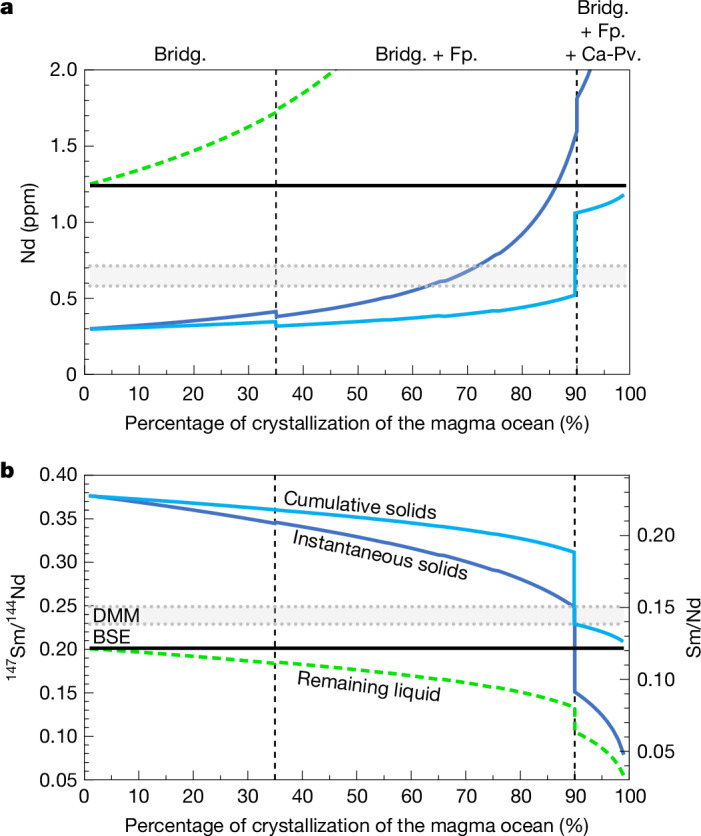
Modelled ^147^Sm/^144^Nd ratios and Nd contents in the liquid and solid phases during terrestrial magma ocean crystallization in deep-mantle conditions. **a**, Nd contents. Bridg., bridgmanite; Ca-Pv., Ca-perovskite; Fp., ferropericlase. **b**, ^147^Sm/^144^Nd ratios. Dashed green lines indicate liquid phase and solid blue lines indicate solid phase. Their evolution follows the crystallization sequence described in Methods, deriving from ref. ^35^ and using a non-chondritic BSE starting composition^38^. The compositions of the instantaneous solids formed throughout the crystallization process (dark blue) and of the cumulative solids (light blue) are both shown. The kinks at 35% and 90% are artefacts owing to the calculation method (Methods). The compositions of a non-chondritic BSE^38^ and DMM^27,41^ are also shown for comparison.

Here we explore the possibility that magma ocean solidification in the deep mantle could have produced and preserved such a reservoir throughout Earth’s history. It took place in the aftermath of the Moon-forming giant impact, when the mantle was extensively molten down to the core–mantle boundary^31^. Recent petrological, mineralogical and dynamical models all support the formation of crystals in the deep mantle and a basal magma ocean^32–34^. Under these conditions, solidification follows a three-stage sequence^35,36^: (1) bridgmanite crystallizes alone until 30–40% solidification^35,36^; (2) bridgmanite and ferropericlase crystallize together until roughly 90% solidification; and (3) bridgmanite, ferropericlase and Ca-perovskite crystallize for the final few percent of solidification (Fig. 2; details in Methods). We calculated the trace-element composition of the crystallizing solids throughout this sequence using a starting liquid with either a chondritic primitive mantle REE content^37^ or a non-chondritic mantle composition^38^ (Methods). Because bridgmanite is the first mineral to crystallize in a deep magma ocean^35^ and remains so until more than one-third of the mantle has crystallized^36^, its formation will take place in the earliest stages of solidification. It exerts the greatest influence on the trace-element distribution between primitive melts and solids, as well as their isotopic signature. For that reason, we directly measure the Sm and Nd partition coefficients between bridgmanite and melt by performing experiments using a laser-heated diamond anvil cell (DAC) at pressures ranging from 53 to 97 GPa (details in Methods), yielding D_liq–brid_(Nd) = 0.24 ± 0.12 and D_liq–brid_(Sm) = 0.45 ± 0.10 under deep-mantle conditions. Less critical in our models, the Nd and Sm partition coefficients between Ca-perovskite and melt and between ferropericlase and melt are taken from lower-pressure experiments in the literature^39,40^. Figure 2 illustrates the evolution of the Nd content and the Sm/Nd ratio in both the liquid and solid phases during magma ocean crystallization in a non-chondritic scenario, using these parameters. Two types of solid are represented: instantaneous solids, which best describe the chemical heterogeneities in the crystallized magma ocean, and cumulative solids, which reflect the average composition of the solids formed up to a given percentage of crystallization. As crystallization progresses, Nd becomes increasingly concentrated in both the residual liquid and the solid phases, whereas the Sm/Nd ratio decreases in both phases (Fig. 2a,b). Up to 90% crystallization of the magma ocean (when Ca-perovskite starts to crystallize), all solids have a minimum Nd content of approximately half that of the DMM^27,41^ (0.3–1.6 ppm; Fig. 2a) but their ^147^Sm/^144^Nd ratio is much higher (between 0.38 and 0.25; Fig. 2b). Between 90% and 100% crystallization, the Nd content, in both solids and liquids, increases substantially and the ^147^Sm/^144^Nd ratio drops below the initial liquid composition. It should be noted that previously published Sm and Nd partition coefficients between bridgmanite and liquid^39^ are much lower and their use would result in much lower Nd contents in the solid phase. However, the Sm/Nd ratios would remain comparable. The results are essentially identical in a chondritic Earth scenario (see details in Methods). We therefore propose that most of the deep mantle that crystallized from the magma ocean was dominated by bridgmanite and had a high Sm/Nd ratio. This makes it an ideal candidate for producing mantle material with positive ^142^Nd anomalies.

## Deep origin of positive ^142^Nd anomalies

**Fig. 3:**
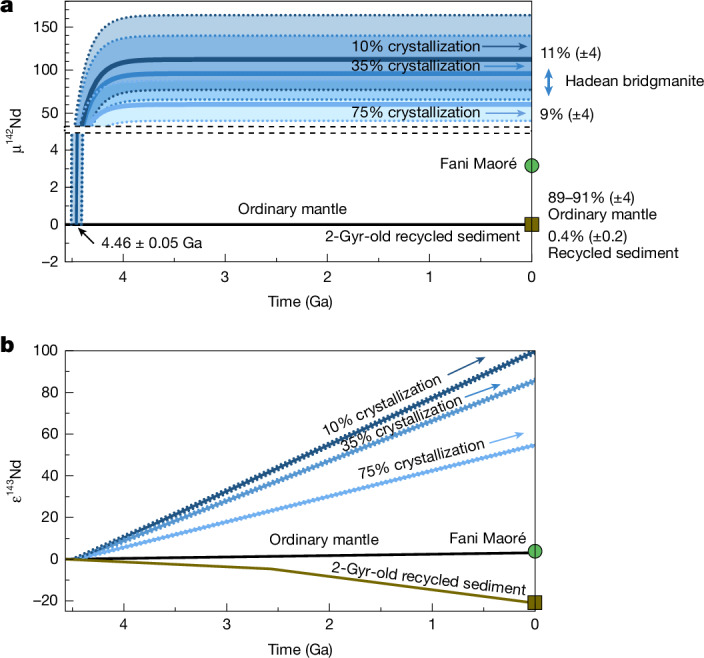
Evolution model of the µ^142^Nd anomaly and the ε^143^Nd composition of the Hadean bridgmanite and a recycled crustal reservoir. **a**, Evolution of the µ^142^Nd anomaly. **b**, Evolution of the ε^143^Nd composition. Hadean bridgmanite is indicated in blue and a recycled crustal reservoir is shown in brown. The mean value of Fani Maoré lavas is shown as a green circle and the ordinary mantle taken here as the non-chondritic BSE of ref. ^38^ as a black line. The present-day isotope composition of the Hadean bridgmanite corresponds to the range of possibilities when considering 10–75% crystallization of a deep terrestrial magma ocean at 4.46 ± 0.05 Ga: at 10% crystallization, µ^142^Nd = +112 (+52/−35) and ε^143^Nd = +100 (±1), whereas at 75% crystallization, µ^142^Nd = +60 (+28/−19) and ε^143^Nd = +55 (±1). The modelled recycled crustal component is assumed to be 2-Gyr-old recycled sedimentary material and is calculated using the GLOSS-II estimate of ref. ^50^ and the same equations as in ref. ^52^. A chondritic mantle scenario would yield similar results but with small differences of 2–6 ppm for both µ^142^Nd and ε^143^Nd values.

The present-day Nd isotopic composition of solids that crystallized at depth from an early terrestrial magma ocean can be modelled for a range of instantaneous solids, assuming an age for their crystallization. Here we consider the giant impact that formed the Moon to be the last event that melted most of the terrestrial mantle^31,42^, generating the last major magma ocean. This event would have erased any previous heterogeneities^43^ and, although its timing is unclear, it is estimated to have occurred either within the first 65 Myr of Earth’s history^44^ or up to 80 Myr later^45^. Here we use an age of 4.46 Gyr (refs. ^46,47^), as suggested by the age of the oldest lunar material, but we account for notable uncertainties by applying an error bar of 50 Myr. Indeed, the timescale of crystallization of a terrestrial magma ocean is not well constrained, with estimates ranging from thousands^48^ to millions^49^ of years. We selected three snapshots during magma ocean solidification (10%, 35% and 75% of crystallization; Fig. 3) to investigate the isotopic evolution of the solid phases. These correspond to a magma ocean that has solidified 10% of its mass and only crystallized bridgmanite and then 35% and 75% of its mass, crystallizing a mixture of bridgmanite and ferropericlase. In all of these cases, the Sm/Nd ratio of the instantaneous solid is much higher than that of both the BSE or a mantle depleted by crust extraction. The modelled present-day µ^142^Nd values range from +112 at 10% crystallization to +60 at 75% (Fig. 3a), with corresponding ε^143^Nd values ranging from +100 to +55 (Fig. 3b). The contribution of such material in the source of Fani Maoré lavas could explain the measured positive ^142^Nd anomalies.

Recent geodynamic simulations have shown that a fraction of the earliest formed solids can be preserved over time during Hadean magma ocean crystallization and subsequent mantle convection^32^. Therefore bridgmanite-rich material in the Hadean deep mantle could have been preserved in a convecting, ordinary mantle, contributing to the Fani Maoré source, alongside the present-day ordinary mantle (µ^142^Nd = 0 and ε^143^Nd = +3.07, assuming a non-chondritic silicate mantle^38^). The mean µ^142^Nd at +3.2 of Fani Maoré can be reproduced by including 8–10(±3)% of Hadean bridgmanite in the source, but reproducing its mean ε^143^Nd of +3.8 would require only 2–3(±1)%. This demonstrates that a simple mixture of Hadean material and ordinary mantle cannot explain all of the observations and that a third component is needed in the source of Fani Maoré volcanics. Numerous studies have suggested that sedimentary material is often involved in the source of OIB (see recent review^1^). To investigate whether the presence of recycled sediments in the source could reconcile the constraints provided by ^142^Nd and ^143^Nd isotopes, we modelled the impact of a typical subducted sediment, global subducting sediment (GLOSS-II)^50^, with an estimated age of 2 Gyr being involved in the mixed source. This material is young enough to have a µ^142^Nd of 0, yet it exhibits a highly negative ε^143^Nd of about −21 (Fig. 3). Adding about 0.4% sedimentary material barely alters the proportion of Hadean bridgmanite (9–11% instead of 8–10%) and reconciles the constraints provided by µ^142^Nd and ε^143^Nd (see Fig. 3 and details in Methods). This is also consistent with the Sr and Pb isotopic compositions of the lavas^51^. It should be noted that the exact proportion of Hadean material necessary to reproduce the µ^142^Nd of Fani Maoré depends on the crystallization age of the magma ocean. However, it remains within a small range as long as the crystallization did not occur after 4.35 Ga, when the decay of ^146^Sm produced little ^142^Nd.

## Implications for mantle dynamics

**Fig. 4:**
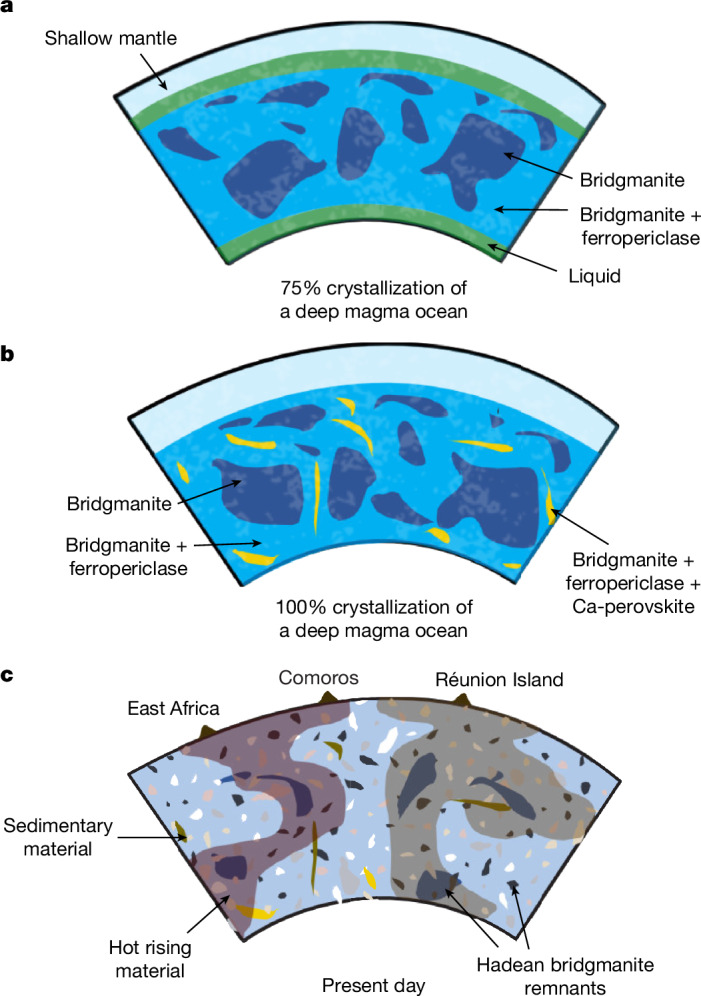
Schematic representation of the crystallization of a deep magma ocean followed by mantle homogenization through convection. **a**, The Hadean bridgmanite-rich material (dark and light blue) represents solids formed during the first 90% of crystallization and includes bridgmanite and bridgmanite plus ferropericlase. **b**, As crystallization progresses to 100%, the solid assemblage transitions to bridgmanite + ferropericlase + Ca-perovskite (yellow). **c**, The source of volcanism at Fani Maoré in the Comoros archipelago consists mainly of ordinary mantle but also includes about 10% of Hadean bridgmanite-rich material and 0.5% of recycled sedimentary material.

The significantly positive µ^142^Nd of the Fani Maoré volcanics suggests the existence and preservation of remnants of Hadean material in the present-day mantle. We evaluated the possibility of a shallow origin for this reservoir through crustal extraction and presented a new model involving a deeper origin through magma ocean solidification. The shallow model requires the source to contain 28–90% of Hadean material, whereas the deep model only requires 9–11%. Independently, recent geodynamical fluid dynamics simulations of magma ocean crystallization^35^ show the widespread production of geochemical (Sm/Nd and Lu/Hf) heterogeneities during solidification and their vertical scattering throughout the mantle. Such material exhibits a strongly depleted REE pattern and formed through the crystallization of most of the deep mantle during the last magma ocean event (Fig. 4). Considering the extensive share of the deep mantle that was once composed of Hadean bridgmanite, it is plausible that some of it has survived in the mantle to be sampled by modern volcanism. Several occurrences of positive µ^142^Nd were previously reported for Réunion Island^11^, Iceland^12^ or Pitcairn^13^, but their interpretation was limited by analytical precision. Measurement of a large set of OIB with improved precision might establish that similar Hadean material contributes to OIB sources more commonly than previously thought. Regardless of the deep (bridgmanite crystallization) or shallow (crustal extraction) origin of the anomalous source in Fani Maoré, the preservation of such ancient heterogeneities, formed within the first 100 Myr of Earth’s history, highlights the incomplete mixing of the mantle over geological time.

## Methods

### Geological context of the samples

**Extended Data Fig. 1:**
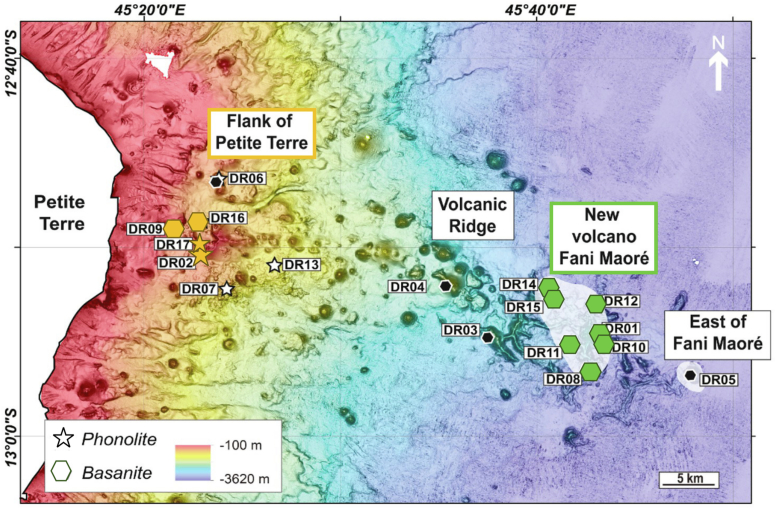
Map showing the location of the samples analysed during this study, modified after ref. ^51^. Mayotte samples were collected from the eastern flank of Petite-Terre and the samples selected for this study are shown as yellow hexagons (basanites) and stars (phonolites). The basanites collected from the new submarine volcano Fani Maoré are represented as green hexagons.

The Comoros archipelago is the surface manifestation of a deep plume. The main islands that make up this archipelago are Grande Comore, Mohéli and Anjouan, as well as the islands that constitute Mayotte, namely Grande-Terre and Petite-Terre, from west to east^53^. Of these, Mayotte corresponds to the oldest volcanic activity, with the first subaerial eruption occurring about 11 million years ago (Ma). However, its volcanic activity has alternated between periods of active and quiescent phases, shaping its two islands of Grande-Terre and Petite-Terre^54^. The most recent volcanic activity was observed on Petite-Terre and is Holocene^54,55^ in age. Between June 2018 and January 2021, a new submarine volcano called Fani Maoré erupted approximately 55 km east of Mayotte, representing the most recent volcanic expression of the plume (see Fig. 1 in ref. ^56^ and Extended Data Fig. 1). The 2018–2021 crisis that led to the formation of Fani Maoré is associated with a volcanic ridge that extends westwards towards Petite-Terre^56,57^. In this context, we analysed 13 samples from the new submarine volcano Fani Maoré and eight samples from the eastern flank of Petite-Terre, Mayotte (Extended Data Fig. 1). These samples were collected during a series of oceanographic cruises between 2018 and 2021 (MAYOBS1, MAYOBS2, MAYOBS4 and MAYOBS15 (refs. ^57,58^)) as part of several dredging operations targeting both the Fani Maoré volcanic edifices and the eastern flank of Petite-Terre. All Fani Maoré samples are basanites, whereas Petite-Terre samples include three basanites and five phonolites. Detailed petrological descriptions can be found in ref. ^56^ and chemical and isotope compositions are reported in ref. ^51^.

### Sample preparation and Nd isotope measurements

All 21 OIB samples were processed in the clean laboratory of the Institut de Physique du Globe de Paris (IPGP) and their Nd isotope compositions were measured on a Nu Instruments TIMS, following procedures described in ref. ^22^. Depending on the REE concentration and the amount of powder available, aliquots of 50–300 mg of homogenized bulk rock powder were digested in a 3:1 mixture of distilled 28 M HF and 15 M HNO_3_ at 75 °C for 48 h on a hotplate and then evaporated to incipient dryness. Fluorides were decomposed with repeated dissolution and evaporation cycles using alternating 6 M HCl and a 1:1 mixture of 6 M HCl and 15 M HNO_3_, until the solution was clear of any precipitate after centrifugation. Neodymium was chemically isolated using a four-step chemical separation procedure. REE were initially separated from the matrix by cation exchange chromatography using AG50W-X8 resin (200–400 mesh; 2 ml for a typical sample of 35–50 mg). Samples with digested mass exceeding 50 mg were divided into several columns to avoid saturation of the resin. The resulting REE fractions were subsequently recombined. In the second step, Ce was separated from the other REE using a redox technique and LN resin (50–100 µm; 0.5 ml). Sodium bromate (NaBrO_3_) was used to oxidize Ce from Ce^3+^ to Ce^4+^, allowing it to be retained in the LN resin while the other REE^3+^ were eluted. This step was completed twice to ensure complete Ce removal. Residual Na and Br were removed from the Ce-free REE fraction during the third step using AG50W-X8 resin (200–400 mesh; 1 ml). Finally, in the fourth step, Nd was isolated from the other REE using a thin LN resin (25–50 mesh; 0.82 ml). Total procedural Nd yields were ≥90% and the procedural blank for Nd was less than 40 pg (*n* = 3), which is negligible relative to the 1–9 µg of Nd collected from the samples. Residual Sm and Ce remaining in the final Nd fraction were consistently negligible (<0.5 ng Sm and <0.2 ng Ce) relative to the mass of Nd.

High-precision multi-dynamic Nd isotope analyses were performed on a Nu Instruments TIMS at IPGP following a five-line acquisition method thoroughly detailed in ref. ^22^ (cup configuration in their Fig. 1). For both standards and samples, about 800 ng of Nd per analysis was loaded onto zone-refined 99.999% Re filaments used in a double-filament configuration. The five-line acquisition method in positive mode allows the simultaneous measurement of all Nd isotopes during all five acquisition lines, each separated by a one-mass-unit jump. This technique allows the combination of two to three acquisition lines to dynamically measure (in the same Faraday cup) three ratios per Nd isotope, the average of which gives a multi-dynamic ratio essentially free from biases owing to the Faraday cups. A typical run consists of 40 blocks of 20 cycles with about 6 V of ^142^Nd^+^ measured for 18 h. Mass discrimination was corrected using ^146^Nd/^144^Nd = 0.7219 and the exponential law. Time drift was corrected using 11-cycles interpolations. Data were systematically corrected^22^ for Sm and Ce isobaric interferences in case ^147^Sm/^144^Nd was higher than 7 × 10^−7^ or ^140^Ce/^144^Nd was higher than 9 × 10^−6^. This allows reliable correction and prevents overcorrection that might increase errors. The detection threshold to consistently distinguish Sm and Ce signal from the background noise during our analyses is established at ^147^Sm/^144^Nd > 5 × 10^−7^ and ^140^Ce/^144^Nd > 5 × 10^−6^ (for I^142^Nd^+^ about 5 V; see Fig. 9a,c in ref. ^22^).

**Extended Data Fig. 2:**
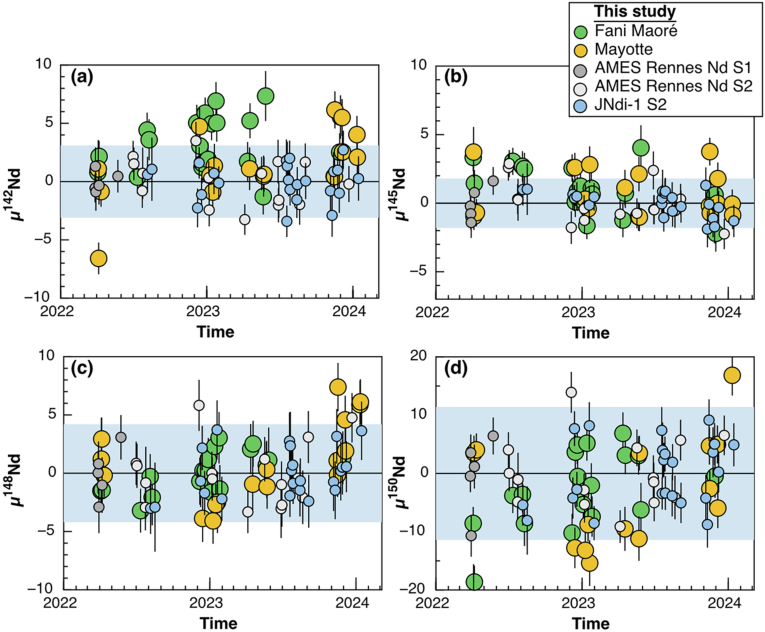
Nd isotope measurements of Fani Maoré and Mayotte samples, alongside AMES Rennes Nd and JNdi-1 reference materials. **a**, µ^142^Nd. **b**, µ^145^Nd. **c**, µ^148^Nd. **d**, µ^150^Nd. The measured Nd isotope ratios are reported in the mu notation versus their date of acquisition. Error bars are 2 s.e. and the blue bands represent the 2 s.d. on JNdi-1.

**Extended Data Fig. 3:**
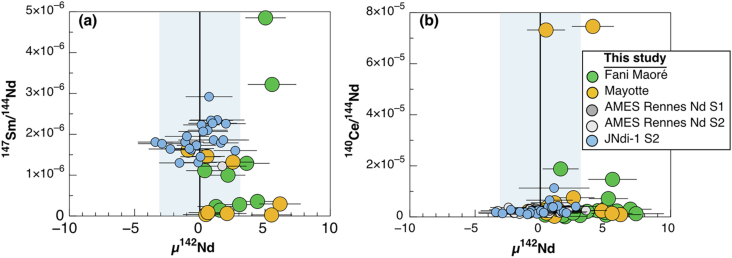
Monitored Sm and Ce interferences compared with the µ^142^Nd of Fani Maoré and Mayotte sample measurements, alongside AMES Rennes Nd and JNdi-1 measurements. **a**, Sm. **b**, Ce. Error bars are 2 s.e. and the blue band represent the 2 s.d. on JNdi-1.

External precision was evaluated using two pure Nd solution standards measured over two analytical sessions, alongside the samples. We analysed AMES Rennes Nd five times during the first session (March 2022 to May 2022) and we analysed AMES Rennes Nd and JNdi-1 15 and 23 times, respectively, during the second session (July 2022 to January 2024). They gave similar isotope ratios within error, with typical reproducibility (2 s.d.) between 1.8 and 3.9 ppm for ^142^Nd/^144^Nd, 2.0–3.8 ppm for ^143^Nd/^144^Nd, 1.8–3.1 ppm for ^145^Nd/^144^Nd, 4.2–5.6 ppm for ^148^Nd/^144^Nd and 11.4–11.9 for ^150^Nd/^144^Nd. JNdi-1 was the most frequently measured standard during this study and its reproducibility (2 s.d. of 3.1 for ^142^Nd/^144^Nd) is taken as representative of the external error for all samples. The short-term reproducibility, which is usually reported in the literature as being equivalent to uninterrupted series of samples and standard measurements, is similar. We identify six series (March–May 2022; July 2022; December 2022 to January 2023; April–June 2023; August–September 2023; November 2023–January 2024), the reproducibility of which on the ^142^Nd/^144^Nd ratio ranges from 1.8 to 4.1 ppm (*n* = 4–9, excluding *n* ≤ 3), giving an average reproducibility of 3.1 ppm. Details on the individual measurements of AMES Rennes Nd and JNdi-1 are given in Supplementary Data Table 1b and are shown in Extended Data Figs. 2 and 3.

Rock reference materials were measured during the same analytical sessions as our samples. The results were extensively described in ref. ^22^ and show similar reproducibility.

The ^143^Nd/^144^Nd ratios are given in the epsilon notation following the equation ε^143^Nd = ((^143^Nd/^144^Nd_sample_/^143^Nd/^144^Nd_CHUR_) −1) × 10^4^, in which CHUR is the CHondritic Uniform Reservoir with ^143^Nd/^144^Nd = 0.512630 (ref. ^59^). All other Nd isotope ratios are given in the mu notation following the equation µ^x^Nd = ((^x^Nd/^144^Nd_sample_/^x^Nd/^144^Nd_terrestrial reference_) −1) × 10^6^, in which the terrestrial reference composition for this study is the widely used JNdi-1 pure Nd solution standard. All of the samples analysed during session 2 were measured concurrently with JNdi-1 and their mu values were calculated directly. However, three samples were measured during session 1. To ensure proper comparison of these data with the rest of the dataset, their measured isotope ratios were first normalized to session 2 using the AMES Rennes Nd measured during both sessions, following the equation ^x^Nd/^144^Nd_corrected sample_ = ^x^Nd/^144^Nd_measured sample_ × (^x^Nd/^144^Nd_AMES,session 2_/^x^Nd/^144^Nd_AMES,session 1_). The mu values for these corrected samples were then calculated similarly to the rest of the dataset. All Nd isotope measurements of samples and pure Nd reference materials acquired during the course of this study are reported in Supplementary Data Table 1 and Extended Data Figs. 2 and 3.

### Critical evaluation of the data obtained on natural samples

Under terrestrial conditions, excesses and deficits in ^142^Nd reflect the decay of ^146^Sm. They are subtle and therefore more prone to analytical bias and misinterpretation. To detect these, Sm and Ce interferences as well as ^145^Nd/^144^Nd, ^148^Nd/^144^Nd and ^150^Nd/^144^Nd ratios were precisely measured and monitored.

Samarium and Ce interferences were essentially non-existent for most samples. Nevertheless, six measurements were corrected for an approximate 0.5 ppm Sm contribution and DR1402 for a 2.0 ppm contribution on ^142^Nd/^144^Nd (Supplementary Data Table 1a). Similarly, Ce interference was negligible during most measurements, with only three requiring corrections, with contributions of 1.6 ppm (DR140401) and 6–8 ppm (both measurements of DR070202) on ^142^Nd/^144^Nd. Interference-corrected analyses are unrelated to the extreme ^142^Nd/^144^Nd ratios reported in this study (Extended Data Fig. 3).

**Extended Data Fig. 4:**
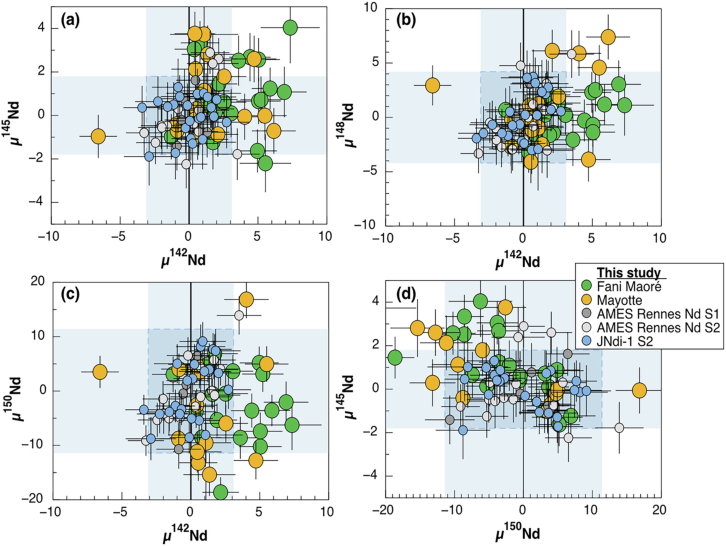
Nd isotope composition of Fani Maoré and Mayotte sample measurements, alongside AMES Rennes Nd and JNdi-1 standard measurements. **a**, µ^145^Nd versus µ^142^Nd. **b**, µ^148^Nd versus µ^142^Nd. **c**, µ^150^Nd versus µ^142^Nd. **d**, µ^145^Nd versus µ^150^Nd. Error bars are 2 s.e. and the blue bands represent the 2 s.d. on JNdi-1.

**Extended Data Fig. 5:**
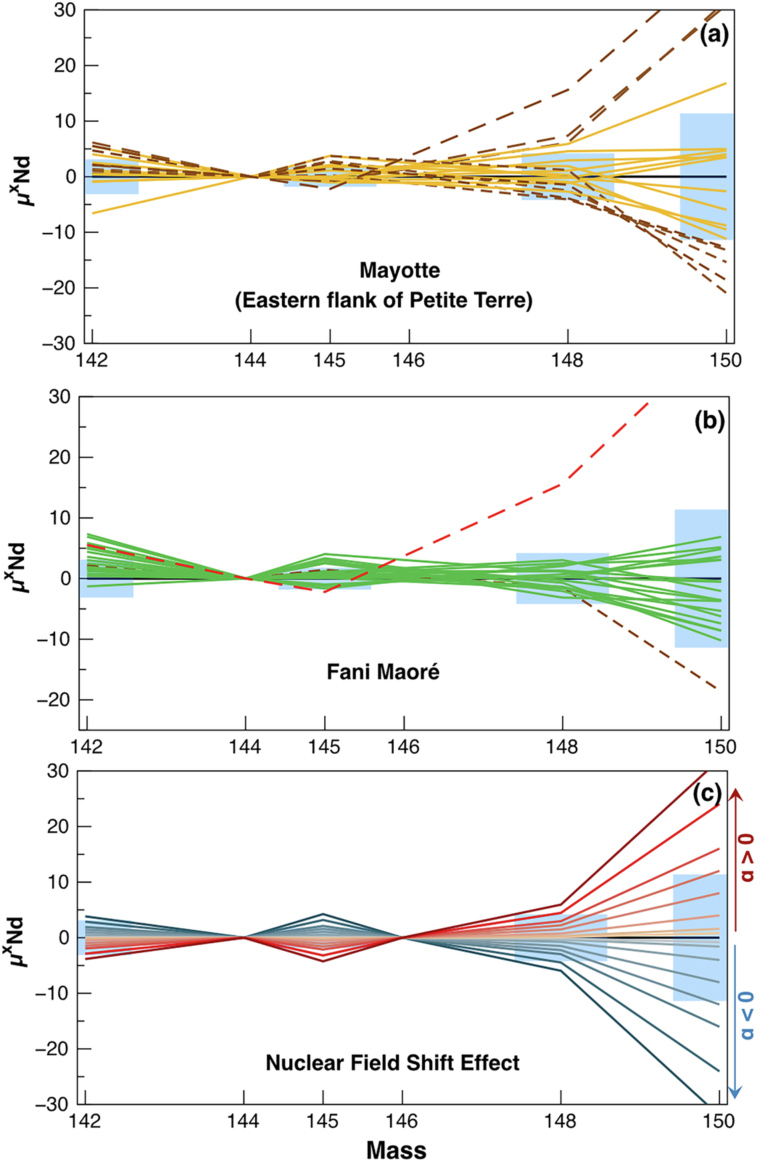
Nd isotope spectra as measured for Mayotte and Fani Maoré samples, along with a theoretical terrestrial material affected by the NSF effect. **a**, Mayotte samples. **b**, Fani Maoré samples. **c**, The NFS effect is calculated using the equations defined in refs. ^60,61^ and published isotope radius and atomic masses for Nd (refs. ^66,81^). The magnitude of Nd isotope variations induced by the NFS effect increases with the intensity of NFS represented by the value *α* (for which increasingly positive or negative values indicate stronger effects).

^145^Nd/^144^Nd, ^148^Nd/^144^Nd and ^150^Nd/^144^Nd ratios are not supposed to show any mass-independent variations in terrestrial conditions. In Extended Data Fig. 4, anomalous variations can be identified, as they may exceed the typical variability of pure standard solutions and can serve as an indicator for potentially biased ^142^Nd/^144^Nd data. In particular, Nd isolation during the chemical procedure can induce mass-independent variations affecting all Nd isotopes^22,23^, called the nuclear field shift (NFS) effect^60,61^. Higher NFS magnitudes lead to larger deviations from terrestrial values (illustrated in Extended Data Fig. 5c). We monitor these Nd ratios for variations exceeding the JNdi-1 reproducibility, indicating possible and associated ppm-level μ^142^Nd deviations. DR1802-1 has ^148^Nd and ^150^Nd excesses (15.6 and 40.4 ppm) and a ^145^Nd deficit (2.2 ppm; Extended Data Table 1), mimicking NFS-induced variations (in red in Extended Data Fig. 5b and Extended Data Table 1). This suggests that its measured positive µ^142^Nd might be underestimated and should be even more positive. However, the lack of convergence in the magnitude of NFS across isotopes prevents us from performing a correction of the measured µ^142^Nd. Excluding DR1802-1, the average Fani Maoré value remains consistent, as it only changes from 3.2 ± 0.9 (2 s.e., *n* = 13) to 3.0 ± 0.9 (2 s.e., *n* = 12). Several other measurements show small excesses or deficits in ^148^Nd and ^150^Nd but no ^145^Nd deviation (in brown in Extended Data Fig. 5a,b and Extended Data Table 1). They do not show a clear pattern resembling fractionation owing to the NFS effect and their ^142^Nd can be considered reliable.

**Extended Data Fig. 6:**
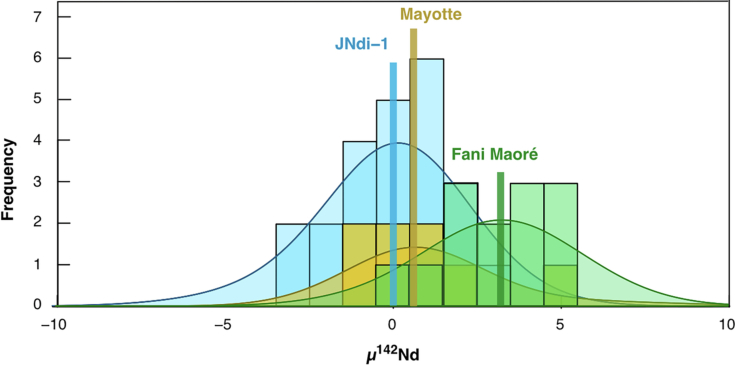
Histogram showing the distribution of Fani Maoré and Mayotte samples, compared with the distribution of JNdi-1 measurements. Fani Maoré samples are indicated in green, Mayotte samples in yellow and JNdi-1 measurements in blue. Histograms are shown alongside their kernel density estimations curves, in green, yellow and blue, respectively (calculated using IsoplotR).

The significance of the ^142^Nd isotope anomaly measured in Fani Maoré or Mayotte samples can be assessed by comparing the lavas dataset with the reference material values and distributions. The 2 s.d. of the mean for all Fani Maoré and Mayotte samples are 3.3 and 3.7 ppm, values that are similar to the standard deviation of JNdi-1 measurements (3.1 ppm). This implies that the total variability of Fani Maoré and Mayotte samples is small and almost corresponds to the analytical precision alone. Although slightly positive, the mean µ^142^Nd of Mayotte of +1.3 ±1.3/±3.6 (2 s.e./2 s.d., *n* = 8) is clearly within the error of JNdi-1. By contrast, the mean µ^142^Nd of Fani Maoré of +3.2 ±0.9/±3.3 (2 s.e./2 s.d., *n* = 13) is at the limit of JNdi-1’s reference value. The statistical distribution of both JNdi-1 and Fani Maoré samples demonstrates that they represent three distinct groups: (1) The 2 s.e. on the mean value for Fani Maoré and JNdi-1 do not overlap, as +3.2 ±0.9 (Fani Maoré; *n* = 13) is distinct from 0 ± 0.7 (JNdi-1; *n* = 23). (2) The distinction between the Fani Maoré and JNdi-1 populations is also assessed with a *t*-test, providing a *P*-value of 8.9 × 10^−6^, in which the two populations are considered statistically distinct whenever the *P*-value is below 0.05. By contrast, the *P*-value when comparing Mayotte and JNdi-1 is 0.14, thus showing that they are statistically undistinguishable. (3) The distribution of Fani Maoré samples in histograms also points towards a strict distinction from JNdi-1 (Extended Data Fig. 6), as the maximum of their Gaussian distribution curve is distant by 3.5 ppm from JNdi-1, with 95% of Fani Maoré data located between +2 and +5, whereas 95% of JNdi-1 measurements are located between −2 and +2.

### Relationship to other radiogenic isotopes and trace elements

**Extended Data Fig. 7:**
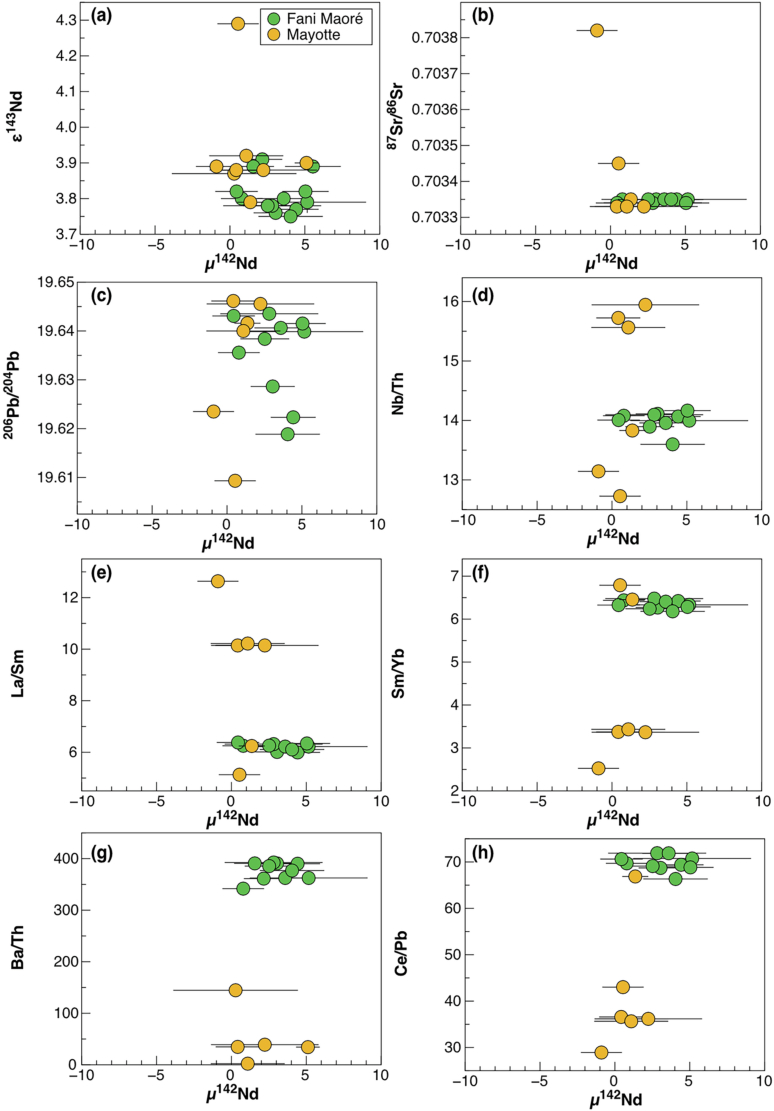
Isotope compositions and trace-element ratios of Mayotte and Fani Maoré samples investigated in this study, compared to their µ^142^Nd value. **a**, ε^143^Νd. **b**, ^87^Sr/^86^Sr. **c**, ^206^Pb/^204^Pb. **d–h**, Trace-element ratios. Sr and Pb isotopes and trace-element compositions are from ref. ^51^. Error bars correspond to 2 s.e. on individual measurements of ^142^Nd/^144^Nd and 2 s.d. when samples were measured several times.

Fani Maoré lavas were previously studied for their radiogenic isotope and trace-element composition^51^. Their radiogenic isotopes are very uniform (see Extended Data Fig. 7 and Supplementary Fig. 2 in ref. ^51^) and lie at intermediate compositions between HIMU to EM-I. Although the Nb/Th ratio does not vary much, basanites and phonolites have somewhat different ratios of elements, such as La/Sm, Sm/Yb, Ba/Th or Ce/Pb. Discussing the origin of the exceptionally high Ba/Th and Ce/Pb ratios is beyond the scope of this manuscript and we encourage the reader to look at the interpretation suggested in ref. ^51^. Here we concentrate on the relationship between these ratios and µ^142^Nd. Extended Data Fig. 7 shows that no clear relationship exists between the presence of a positive ^142^Nd anomaly and any of the above-mentioned radiogenic and trace-element ratios, suggesting that the origin of the ^142^Nd anomaly might not be related to the process that led to the presence of high Ce/Pb and Ba/Th ratios.

### Isotope modelling: assumptions about the composition of the mantle

Estimating the present-day µ^142^Nd and ε^143^Nd composition of a Hadean reservoir requires assumptions about the composition of the mantle after Earth accretion and today. Several studies suggest that the lithophile part of the Earth after core formation (BSE) has a non-chondritic Sm/Nd ratio owing to depletion events occurring within the first few million years of Earth history. Suggestions include: (1) collisional erosion of the early planetesimals that built the Earth^38,62^; (2) heterogeneous mineral distribution in the inner Solar System^63^; and (3) buried ancient crust that left an early depleted reservoir^64^. Despite these different interpretations, all studies converge on a 2.40–2.65% higher Sm/Nd ratio than in chondrites^38,59,63^, yielding a Sm/Nd = 0.333 and ^147^Sm/^144^Nd = 0.2012 (calculated as ^147^Sm/^144^Nd = Sm/Nd × 0.60455923 using Sm and Nd isotope abundances and molar masses^65,66^) and an initial ^146^Sm/^144^Nd calculated at 0.00034703 using an initial Solar System ^146^Sm/^144^Sm of 0.00840 (ref. ^67^), a Solar System age of 4.5674 ± 0.0007 Gyr (ref. ^68^) and a decay constant for ^146^Sm of 92 Myr (ref. ^9^). Following these assumptions, the present-day Nd isotope composition of the BSE is ε^143^Nd = +3.07 and µ^142^Nd = 0, values that we will use as reference for a present-day ordinary mantle. All input and output data used in our models are listed in Extended Data Tables 2 and 3.

### Samarium and neodymium partition coefficients between bridgmanite and melt

To best investigate the trace-element signature of Hadean bridgmanite crystallizing out of a magma ocean, we conducted laboratory experiments reproducing the solidification process under terrestrial conditions. Specifically, we performed fractional crystallization experiments in a laser-heated DAC, following the protocol established in ref. ^36^. This protocol, originally conceived for quantitative major-element concentration measurements in the various phases (minerals along the fractional crystallization sequence as well as the residual melt), has recently been extended to include trace-element measurements at the 100-ppm level^69^. We used it here to determine the partition coefficient of Sm and Nd between bridgmanite and melt under deep-mantle conditions relevant to magma ocean solidification, specifically between 53 GPa at 3,200 K and 97 GPa at 3,700 K (final temperatures). The starting material for our experiments, loaded into the DAC, had a pyrolitic major-element composition (representative of the BSE) and was doped with several trace elements, including 3,000 ppm of Sm and Nd. This material was synthesised by mixing pure oxides and pure trace-element solutions. The mixture was then fused for 60 s at 1,900 °C (above the liquidus) in a gas-mixing aerodynamic levitation laser furnace^70^ to achieve full chemical equilibration^71^ before being rapidly quenched into a glass. The resulting glass was polished and analysed using a field emission gun scanning electron microscope to verify its chemical homogeneity and the absence of crystals; its composition is detailed in Supplementary Data Table 2. The glass was subsequently loaded in a DAC, compressed to the target pressure and melted using a double-sided laser heating system. It was then slowly cooled^36^ (10–30 K s^−1^) to a low residual melt fraction, yielding a fractional crystallization sequence at a set pressure, before being quenched to freeze in the chemistry and mineralogy. After decompression, a thin section was retrieved in the centre of the heated region using the focused ion beam lift-out technique and thinned down to electron transparency (about 100 nm), allowing for analysis on an analytical transmission electron microscope. An FEI Tecnai Osiris, equipped with four windowless energy-dispersive X-ray spectrometers at École Polytechnique Fédérale de Lausanne (EPFL), was used for quantitative chemical mapping. The concentrations of Sm and Nd were measured across the sample, with a particular focus on the most primitive bridgmanite crystals that were the first to form following pyrolite crystallization and that are of principal interest here. The composition of these primitive bridgmanite crystals is given in Supplementary Data Table 2. The molar partition coefficients were derived from the Sm and Nd bridgmanite-to-melt ratio, with uncertainties calculated through classical propagation of those obtained from the chemical composition measurements. Finally, these molar partition coefficients were converted to mass partition coefficients (Extended Data Table 4) for further geochemical modelling, although the difference is negligible (less than 1%) and falls well within the uncertainty, thus it can be disregarded.

Our data can be compared with the data in ref. ^39^, which are multi-anvil experiments that have demonstrably achieved equilibrium (long-duration experiments, in contrast to the shorter runs reported elsewhere). Also, the lithology investigated in ref. ^39^ closely matches ours and, by extension, the BSE (a pyrolite composition doped with trace elements). Our partition coefficients for Sm and Nd are an order of magnitude higher than those measured at 25 GPa and 2,300 °C. This can be expected for several reasons, from thermodynamics and crystal chemistry. Our experiment is at 65 GPa and 4,000 °C. At higher temperature (a natural consequence of increasing liquidus temperature with pressure), element partitioning tends to unity (equipartition principle of statistical physics). At higher pressure, the difference in compressibility and size of the host site for large cations (between crystal and melt) decreases, as expected from lattice strain theory, so that partitioning should also tend towards unity.

### Fractional crystallization modelling

The Nd isotopic composition of solids formed at great depth in the mantle during magma ocean solidification is controlled by the minerals/liquid partition coefficients as given in Extended Data Table 4. It also depends on the initial liquid composition chosen here as the non-chondritic REE contents of the BSE estimated using depletion calculations^72^, non-chondritic Sm/Nd ratio^38^ and an initially chondritic primitive mantle^37^. In deep-mantle conditions (>60 GPa; see Fig. 7 in ref. ^35^), the crystallization sequence^35^ is as follows: (1) 100 wt% bridgmanite during the first 35% of crystallization; (2) 90 to 84 wt% bridgmanite and 10 to 16 wt% ferropericlase between 35% and 90% of crystallization, following a simplified linear increase of ferropericlase, an approximation with minimal effect on our conclusions; (3) between 90% and 100% of crystallization, we model the solidification of the residual melt as a bulk containing similar proportions of ferropericlase and bridgmanite and about 4 wt% Ca-perovskite. Element concentrations in the evolving liquid phase are calculated using *C*_liq_ = *C*_liq,0_ × (1 − *X*)^(*D*^^ − 1)^, in which *C*_liq_ is the element concentration in the liquid, *C*_liq,0_ is the initial element concentration in the liquid, *X* is the crystallized fraction and *D* is the partition coefficient. It is noteworthy that this formulation is not formally correct here, because the previous equation only applies in a system with a constant *D*. Our system has varying mineral proportions and partition coefficients, which necessitates to iteratively and incrementally integrate the batch crystallization equation with a parameterized *D*. However, the difference between these two formulations is negligible in the present case. Element concentrations in the cumulative solid are calculated using *C*_cumulate_ = (*C*_liq,0_ × (*X*^*D*^)/*X*, whereas the element concentrations in the instantaneous solids formed at a given crystallization step are calculated using *C*_instant. sol._ = *C*_liq_ × *D*. Neodymium concentrations and Sm/Nd ratios of liquid and solids are shown in Fig. 2.

### Mixing at present day between Hadean bridgmanite and ordinary mantle ± sediments

We evaluate how the incorporation of a Hadean bridgmanite-rich material in modern mantle can reproduce the mean µ^142^Nd and ε^143^Nd isotope compositions measured on the lavas. During the first 90% of crystallization of the magma ocean, all solids have increased Sm/Nd ratio that can evolve to positive µ^142^Nd. We call those solids ‘Hadean bridgmanite’ because it is bridgmanite that predominantly controls the Nd and Sm budget (Fig. 2 and Extended Data Table 4). The last 10% of crystallization produces solids with low Sm/Nd, leading to potentially negative µ^142^Nd. We calculate the present-day isotopic composition of three different solids formed during magma ocean crystallization, the first one at 10% crystallization and two others at 35% and 75% crystallization (Extended Data Table 3).

The present-day µ^142^Nd isotope compositions of these three solids are calculated using a two-step evolution model, here from a non-chondritic BSE:

1. The ^142^Nd/^144^Nd ratio of the mantle evolves from the Earth initial ratio to its composition at the time of formation of the solid, according to its Sm/Nd ratio and following the decay of ^146^Sm during that period
  with ^146^Sm/^144^Nd_BSE,*t*_ = ^146^Sm/^144^Nd_*i*_ × e^(−*λ*(146Sm) × Δ*t*)^.
2. The ^142^Nd/^144^Nd ratio of the Hadean bridgmanite (denoted HB) solid evolves from the isotope composition of the mantle at its time of formation to the present day, according to its Sm/Nd ratio and following the decay of ^146^Sm during that period

with ^146^Sm/^144^Nd_HB,*t*_ = Sm/Nd_HB_ × (^146^Sm/^144^Nd_BSE,*t*_ × Sm/Nd_BSE_)

and ^146^Sm/^144^Nd_HB,today_ = ^146^Sm/^144^Nd_HB,*t*_ × e^(−*λ*(146Sm) × Δ*t*)^.

Values are calculated using a crystallization age of 4.46 ± 0.05 Ga. We also choose the instantaneous solid compositions because they better capture the potential heterogeneities present in the solidified magma ocean. The highly radiogenic Nd isotope composition of the Hadean bridgmanite, coupled with its moderately low Nd content, implies that about 10% of such material is sufficient to reproduce Fani Maoré µ^142^Nd (Extended Data Table 3). However, the same mixture of mantle and Hadean bridgmanite does not reproduce the observed ε^143^Nd of Fani Maoré lavas. To match their ε^143^Nd requires a much lower proportion of Hadean bridgmanite in the mixture (Extended Data Table 3). This is because of the highly radiogenic ε^143^Nd of the Hadean bridgmanite.

**Extended Data Fig. 8:**
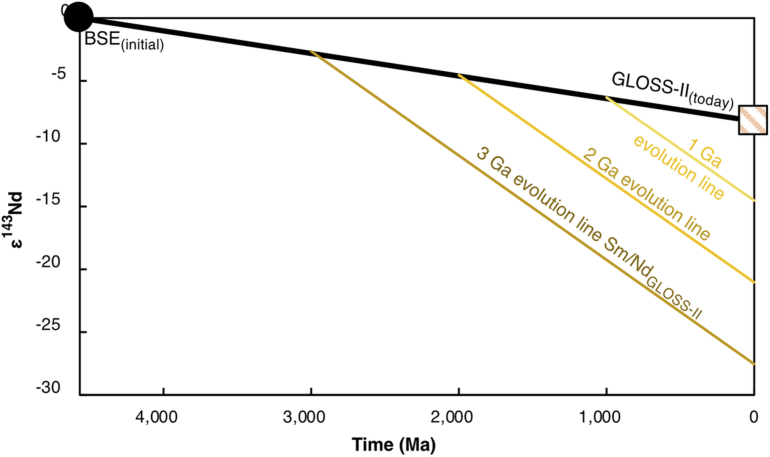
Modelled Nd isotope composition of a recycled surface material with a similar Sm/Nd ratio to GLOSS-II. It is assumed to be recycled into the mantle 1, 2 or 3 Ga, following the recycling model calculations as suggested in ref. ^52^. The initial isotope composition of the recycled surface material at the time of recycling is assumed to be located on the hypothetical line linking the initial isotope composition of the BSE and the present-day composition of GLOSS-II (ref. ^50^). This recycled surface material then evolves to a present-day composition following the Sm/Nd ratio of GLOSS-II (ref. ^50^).

Offsetting the high ε^143^Nd of Hadean bridgmanite requires an extra, geochemically enriched, low-Sm/Nd-ratio material and this material should have no µ^142^Nd anomaly. Recycling of crustal material in the mantle is often suggested to explain the wide isotopic variability observed in OIB (refs. ^1,52,73^), making it a plausible source for a further unradiogenic ε^143^Nd component in the source of Fani Maoré. Here we use GLOSS-II, the average composition of subducted sediments suggested in ref. ^50^ and assume that the recycled sedimentary material has an age of 2 Gyr, which implies no µ^142^Nd anomaly. We calculate its present-day Nd isotopic evolution (Extended Data Table 3) using its published ^143^Nd/^144^Nd and Sm/Nd ratios^50^ (Extended Data Table 2) and the recycling model calculations of ref. ^52^, as shown in Extended Data Fig. 8.

Extended Data Table 3 shows the result of a ternary mixture that includes Hadean bridgmanite, ordinary mantle and recycled sediments. Incorporation of <0.5% of such recycled sediment (GLOSS-II) is sufficient for its low ε^143^Nd to balance the high ε^143^Nd of Hadean bridgmanite and reproduce the value measured for Fani Maoré lavas, while not changing the proportions of Hadean bridgmanite and modern mantle components.

### What happens in the case of a chondritic scenario?

Although the non-chondritic composition of the Earth is a long-standing debate, a chondritic BSE composition cannot be entirely ruled out. We therefore calculated comparable isotope evolution and mixing models using a chondritic composition (input compositions in Extended Data Table 2 and results in Extended Data Table 3). In such case, the proportions of Hadean bridgmanite calculated using ^142^Nd and ^143^Nd are less different from in the non-chondritic BSE scenario when considering a simple mixture of Hadean bridgmanite and ordinary mantle. In this context, no recycled sediments are required in the source (Extended Data Table 3).

### What about a Hadean depleted mantle formed through continental crust extraction?

The range of ^142^Nd/^144^Nd isotopes in Archaean rocks^10,14,25^, together with the Hf isotope variability of zircons^10,74^, indicates the coexistence of both enriched and depleted reservoirs at that time. These anomalies have been attributed either to early crustal extraction producing a depleted mantle residue^14,25,26^ or to large-scale processes generating heterogeneities^11,75,76^. However, there is no clear evidence for large volumes of Hadean crust^77,78^ and only rare evidence of mantle depletion before about 3.8 Ga (ref. ^28^); most crustal growth models also favour progressive rather than massive early extraction^79^. Nevertheless, here we test whether the formation of a Hadean crust could create a suitable depleted Hadean mantle reservoir.

**Extended Data Fig. 9:**
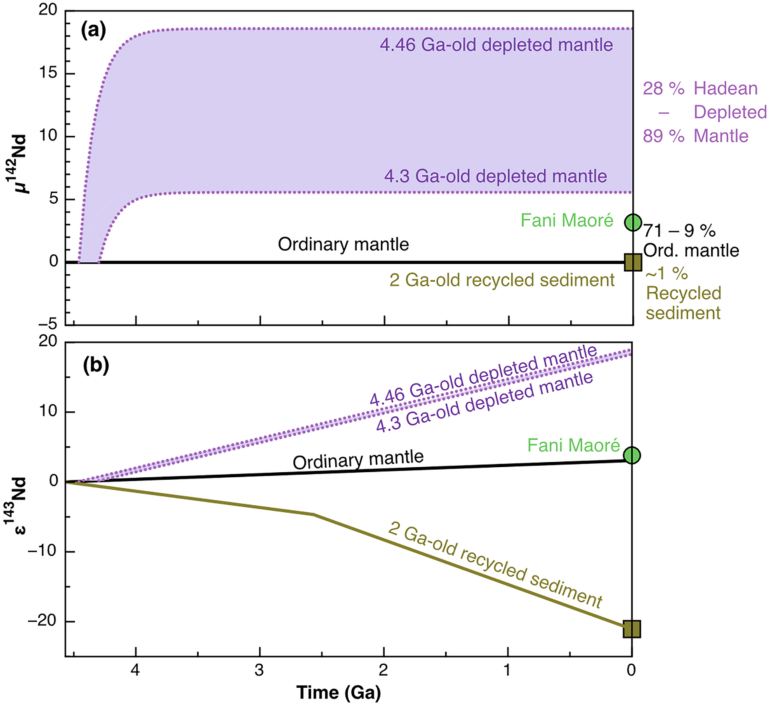
Evolution model of the µ^142^Nd anomaly and the ε^143^Nd composition of a Hadean depleted mantle formed between 4.46 and 4.3 Ga and a recycled crustal reservoir. **a**, Evolution of the µ^142^Nd anomaly. **b**, Evolution of the ε^143^Nd composition. Hadean depleted mantle is indicated in purple and a recycled crustal reservoir is shown in brown. The mean value of Fani Maoré lavas is shown as a green circle and the ordinary mantle taken here as the non-chondritic BSE of ref. ^38^ as a black line. The modelled recycled crustal component is assumed to be 2-Gyr-old recycled sedimentary material and is calculated using the GLOSS-II estimate of ref. ^50^ and the same equations as in ref. ^52^. A chondritic mantle scenario would yield similar results but with small differences of 2–6 ppm for both µ^142^Nd and ε^143^Nd values.

Because crust extraction processes could not be very different from today’s processes, it is expected that a Hadean reservoir depleted by crustal extraction would be similar to the present-day DMM. Here we assume that its Sm/Nd ratio is the same as the DMM in ref. ^27^ (Extended Data Table 2). To observe significantly positive µ^142^Nd in this reservoir, it must form during the first 300 Myr of the Earth’s history. For a depletion age of 4.46 Gyr, the depleted reservoir has a µ^142^Nd of +19 and for a depletion age of 4.3 Gyr, it has a µ^142^Nd of +6 (Extended Data Fig. 9). Using these values and the Nd concentration of DMM^27^, we can calculate how much material is required to match the Fani Maoré µ^142^Nd value. If the material is 4.46 Gyr old, it must represent 26% of the plume source, and if it is 4.3 Gyr old, it must represent 70% of the plume source. However, similarly to the Hadean bridgmanite scenario, offsetting the high ε^143^Nd of a Hadean depleted mantle requires an extra, geochemically enriched, low-Sm/Nd-ratio material devoid of µ^142^Nd anomaly.

Recycling of a 2-Gyr-old GLOSS-II-like crustal material in the mantle is again a plausible source for a further unradiogenic ε^143^Nd component in the source of Fani Maoré. Extended Data Fig. 9 shows the result of a ternary mixture that includes Hadean depleted mantle, ordinary mantle and recycled sediments. Incorporation of about 1% of such recycled sediment (GLOSS-II) is sufficient for its low ε^143^Nd to balance the high ε^143^Nd of the Hadean depleted mantle and reproduce the value measured for Fani Maoré lavas, but it requires to substantially increase the proportion of Hadean depleted material to 28–90% (for formation ages of 4.46–4.3 Ga). Such scenario requires the depleted mantle owing to crustal extraction to form extremely early after the giant impact. For the source of Fani Maoré to be largely composed of such depleted mantle requires that the Hadean component is large enough to be preserved unaltered for more than 4 Gyr. As it formed in the upper mantle, it needs to be dense enough to sink at the bottom of the lower mantle, where it could potentially escape the efficient stirring owing to convection. The survival of localized Hadean depleted domains originating from the upper mantle thus cannot be ruled out but meeting the conditions for it to have survived up to today in notable amounts makes this option a low-probability scenario.

### Involvement of a Hadean felsic material in the source

Early felsic materials such as Isua noritic dykes have been shown to have a positive μ^142^Nd (ref. ^75^) (≤+20), but because of their low Sm/Nd ratios, they also have very negative ε^143^Nd values^75^ (≈−30). Mass balance calculations demonstrate that the positive μ^142^Nd and ε^143^Nd of Fani Maoré cannot be reproduced by incorporating such material.

## Online content

Any methods, additional references, Nature Portfolio reporting summaries, source data, extended data, supplementary information, acknowledgements, peer review information; details of author contributions and competing interests; and statements of data and code availability are available at 10.1038/s41586-026-10719-w.

## Supplementary information

Supplementary Data Table 1 Static, multi-static, dynamic and multi-dynamic Nd isotope ratios obtained with the five-line acquisition method on the Nu Instruments TIMS during this study.

Supplementary Data Table 2 Composition of the starting material and bridgmanite resulting from DAC experiments performed during this study.

Peer Review File

## Figures and Tables

**Extended Data Table 1:**
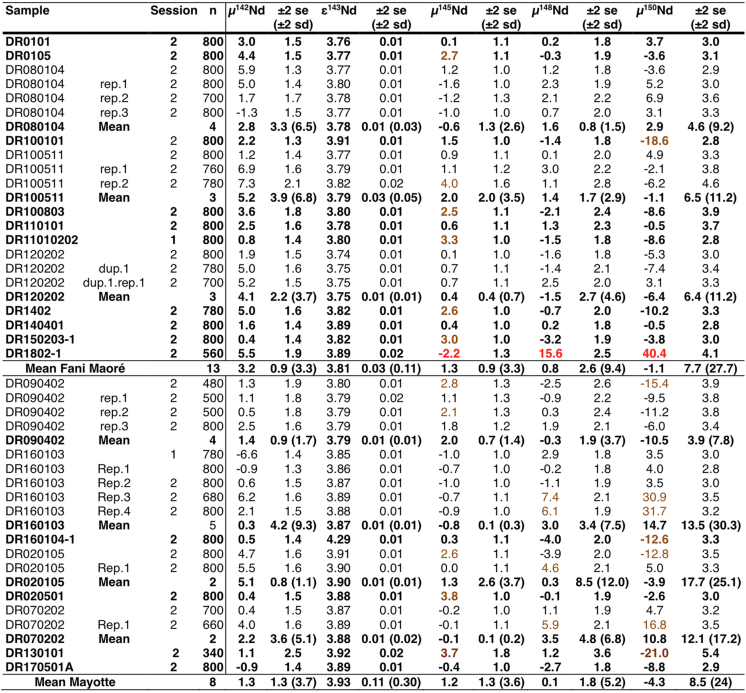
Nd isotope composition of Fani Maoré and Mayotte samples measured during the course of this study

^142,145,148,150^Nd/^144^Nd are reported in the mu notation relative to the mean composition of JNdi-1 measured during session 2 and ^143^Nd/^144^Nd is reported in the epsilon notation relative to the CHUR value^59^. Mean values on Fani Maoré and Mayotte are calculated using the average values for each sample (when available). Reported errors are 2 s.e. for individual measurements and 2 s.e./2 s.d. for the mean values of repeated measurements and for the average values of Fani Maoré and Mayotte.

Values reported in brown show a small excess or deficit in ^145^Nd, ^148^Nd or ^150^Nd, beyond the reproducibility on JNdi-1 Nd isotope measurements, whereas values reported in red show a marked excess that requires attention.

**Extended Data Table 2:**
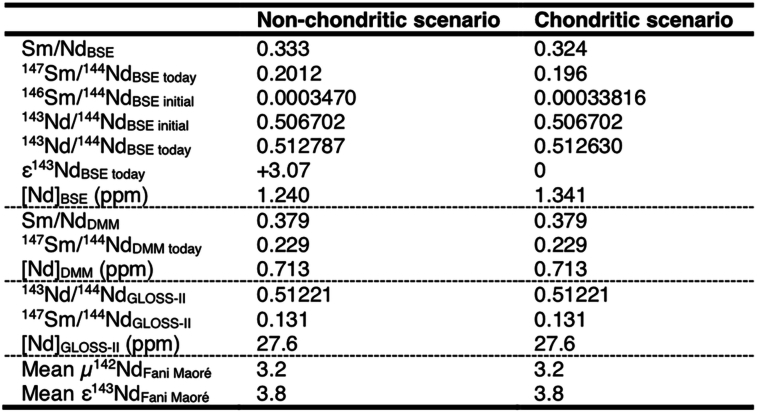
Input parameters

‘initial’ corresponds to an age of 4.5674 Gyr (ref. ^68^). The Sm/Nd ratio of the BSE for the non-chondritic scenario corresponds to the chondritic Sm/Nd ratio^59^ (CHUR^59^) increased by 2.65% (ref. ^38^). The initial ^146^Sm/^144^Nd of BSE is calculated using an initial ^146^Sm/^144^Sm of 0.00840 (ref. ^67^) for the Solar System and molar masses and relative abundances of Sm and Nd (refs. ^65,66^). The Nd content of a non-chondritic BSE derives from depletion calculation^72^, anchored to [Lu] = 0.7083 (ref. ^37^) and using a non-chondritic Sm/Nd ratio^38^. The Nd content of a chondritic BSE is a modelled composition of the primitive mantle deriving from a CHUR composition^37^. The Nd and Sm contents of DMM are from ref. ^27^. The Nd and Sm contents and ^143^Nd/^144^Nd ratio of GLOSS-II are those given in ref. ^50^.

**Extended Data Table 3:**
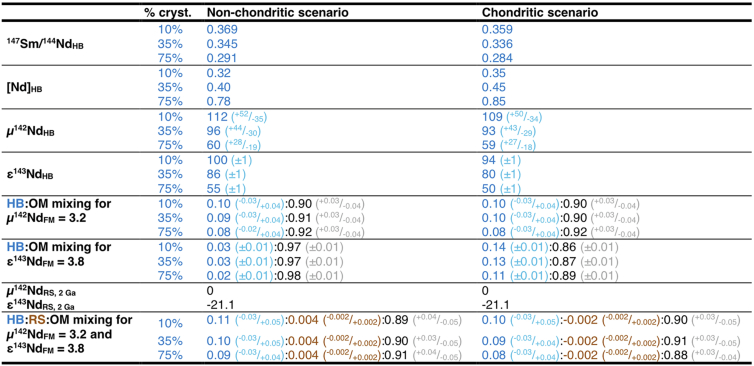
Output parameters

Results for binary and ternary mixing models between an ordinary mantle (OM) taken as a chondritic or non-chondritic BSE, a 4.46 ± 0.05-Gyr-old Hadean bridgmanite-dominated material (HB) and a 2-Gyr-old recycled sedimentary material (RS). The Nd content and isotope composition of the Hadean bridgmanite are calculated using the Sm/Nd ratio of the instantaneous solids at 10, 35 and 75% fractional crystallization and a formation age of 4.46 ± 0.05 Ga (error on results annotated as +0.05 Gyr/−0.05 Gyr). The Nd content and isotope composition of the recycled sedimentary material are calculated using the GLOSS-II Sm/Nd ratio^50^, a recycling age of 2 Gyr and published recycling model calculations^52^.

**Extended Data Table 4:**
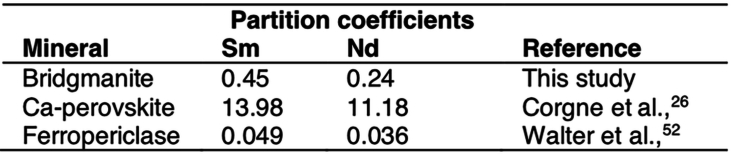
Partition coefficients used in the fractional crystallization models in deep-mantle conditions

Previously published partition coefficients for Sm and Nd in bridgmanite in shallow lower mantle conditions were 0.053 and 0.02 in ref. ^39^.

## Data Availability

Geochemical data that support the findings of this study are available in the paper and its Supplementary Information files and through EarthChem at 10.60520/IEDA/114197 (ref. ^80^).
