# Supplementary material for: Hadean bridgmanite in the source of a present-day ocean island
Source: Nature. 2026 Jul 1;655(8122):389–93. doi: 10.1038/s41586-026-10719-w (PMC13345899; doi:10.1038/s41586-026-10719-w)
Supplement: Supplementary file 3 — Peer Review File [file 41586_2026_10719_MOESM3_ESM.pdf]

nature portfolio

Peer Review File

Hadean bridgmanite in the source of a present-day ocean island

Corresponding Author: Dr Claudine Israel

Any redactions in this file are there to maintain patient confidentiality, the confidentiality of unpublished data, or to remove third-party material.

This file contains all reviewer reports in order by version, followed by all author rebuttals in order by version.

Version 0:

Reviewer comments:

Referee #1

(Remarks to the Author)
Review of paper entitled "Hadean bridgmanite in the source of 1 a present-day ocean island" by Claudine Israel, Catherine Chauvel, James Badro, Hui Chen, C.cile H.bert, Edward Inglis

The authors provide new high-precision 142Nd data on OIB from the Comoros archipelago. The data are the result of an impressive analytical: The authors report a new level of analytical precision that most prior papers, with the exception of the early work reported by Caro (who reported 1sigma=1ppm precision on JNdi). It is particularly impressive and useful that the authors report new mineral-melt partition coefficients for Sm and Nd for bridgmanite.

There has been a lot of interest in identifying 142Nd anomalies preserved in Phanerozoic lavas, as this would show that the products of Hadean silicate differentiation of the Earth have been preserved in the mantle for 4.5 Gyr. Unfortunately, the size of the anomalies—if they exist—are on the order of the precision that can be obtained with modern mass spectrometry. Thus, it remains unclear whether true 142Nd anomalies have been detected in Phanerozoic lavas. The authors employ a new generation of mass spectrometer (a TIMS manufactured by Nu Instruments) to push the limits of analytical precision a report a new level of precision that, if demonstrated to be correct, makes this a paper that is suitable for publication in Nature.

However, there are some key issues that the authors will need to deal with before this manuscript is ready for primetime:
1. To better evaluate (and gain more confidence in) the µ142Nd analyses, it would help to see plots that show whether they correlate with with 143Nd/144Nd, with Pb isotopes, and/or with trace element rations (i.e., La/Sm, Nb/Th, Ce/Pb, Sm/Yb, etc etc)? A suite of plots could go into the supplement and would take the authors perhaps 2 hours to make. If the lavas with anomalous 142Nd are somehow different geochemically, then this would be useful to see.
2. The authors attribute the 142Nd to early Sm/Nd fractionation by bridgmanite precipitation from a terrestrial magma ocean. Thus, they invoke a lower mantle process. But I don't understand how the authors rule out depleted domains that formed in the Hadean? These depleted domains need not be volumetrically large. They just need to have formed early—to generate high 142Nd/144Nd—and be preserved. There is ample evidence from the Hadean zircon record for early (Hadean) depleted mantle domains with superchondritic eps176Hf. Perhaps there wasn't a DMM domain exactly as we know it today, but Hadean zircons tell us that there were geochemically depleted mantle domains formed by continental crust extraction. Crust extraction from the upper mantle in the Hadean resulted in depleted domains. Whether you call them DMM or depleted domains doesn't really matter—both form by crustal extraction. The key here is that shallow mantle depleted domains formed by crustal extraction in the Hadean, so the depleted domain with 142Nd/144Nd that the authors report need not come from the deep mantle (i.e., Bridgmanite). I don't think the authors have excluded the possibility of an upper mantle origin for high 142Nd/144Nd observed in the Comores hotspot lavas.
3. It's important for the reader to be able to evaluate fractionation. This is most easily done by providing plots of µ142Nd vs µ145Nd, µ142Nd vs 148Nd, µ142Nd vs µ150Nd. Make 3 panels (µ142Nd vs µ145Nd, µ142Nd vs 148Nd, µ142Nd vs µ150Nd) and plot 4 different data groups in the plots using different symbols: JNdi analyzed during the study, Ames analyzed during the study, all Fani Maoré analyzed during the study, and all Mayotte analyzed during the study. Please show 2SE error bars on all the analyses. This is easy to do—a 30-minute job for the authors--and will help evaluate fractionation influences are at work, thus lending more confidence in their work. Please include these plots in the

supplement as other readers will want to see the data presented this way.
4. It will be important to show all analyses of µ142Nd, µ145Nd, 148Nd, and µ150Nd analyses vs time. Thus, there will be 4 panels (µ142Nd, µ145Nd, 148Nd, and µ150Nd analyses vs time). In each panel show 4 different symbols: JNdi, Ames, and the two different Comoros volcanoes. Please include these plots in the supplement, as other readers will want to see the data presented this way.

Below I provide additional specific comments that should be useful for the authors as they revise the manuscript:

Line 41. High 3He/4He in Hawaii lavas 41 indicates that their source contains primordial mantle material4.
Comment: Would be worth saying that high “3He/4Hd in OIB”, instead of just Hawaii. This is because other OIB—Iceland, Samoa, Galapagos—have 3He/4He that is similar to or higher than Hawaii.

Line 50. Many Archean samples **preserved in continental settings** display excesses and deficits in 142Nd up to 20 parts per million or ppm (see compilation in 10) testifying of the active decay of 146Sm.
Comment: To distinguish from the mantle, and to engage broader leadership, add the suggested text to this sentence.

Line 54. Several studies have sought to identify 142Nd isotope variations in modern MORB and OIB.
Comment: This is a great place to cite proposed anomalies measured in phanerozoic lavas, which are cited elsewhere in the paper.

Line 83. Today, this depleted material 82 would have a µ142Nd of +19 to +4 if 83 formed within the first 100 to 300 million years of Earth’s history.
Comment: explain how you arrived at this range in µ142Nd.

Line 84. To reproduce the µ142Nd of 84 +3.2 measured at Fani Maor. would require up to 80% of such Hadean depleted material mixed 85 up with ordinary mantle to form the source of the volcano.
Comment: 80% assumes a particular value in the range of µ142Nd (+19 to +4) that you provided. Which value did you assume? Also, this percentage requires assumptions about the source concentrations of normal mantle and the Hadean depleted material, but you did not provide the concentrations.

Line 86. Such scenario seems quite unlikely 86 because it would suggest that a depleted mantle similar to the modern DMM was formed as 87 early as the Hadean.
Comment: There is a logical step missing here. The authors assume that the mixing had to happen in the Hadean, but there is no requirement for this. Mixing between the Hadean depleted material and post-Hadean DMM could occur after the Hadean. For example, why couldn’t Hadean depleted material and mix with Proterozoic DMM during the Phanerozoic? Or Hadean depleted material and mix with Phanerozoic DMM during the Phanerozoic? And so forth?

Line 86. Such scenario seems quite unlikely 86 because it would suggest that a depleted mantle similar to the modern DMM was formed as 87 early as the Hadean. This implies a very high crustal extraction rate which seems to be difficult 88 to reconcile with current models of crustal growth16. An alternative explanation involves the 89 creation, within the Hadean mantle, of enriched and depleted domains producing both positive 90 and negative µ142Nd values with time.
Comment: I don’t understand why depleted domains couldn’t form in the Hadean? These depleted domains need not be volumetrically large. They just need to have formed early and be preserved. There is ample evidence from the zircon record for early (Hadean) depleted mantle domains with superchondritic eps176Hf. Perhaps there wasn’t a DMM domain exactly as we know it today, but Hadean zircons tell us that there were geochemically depleted mantle domains formed by continental crust extraction. Crust extraction in the Hadean resulted in deplted domains. Whether you call them DMM or depleted domains doesn’t really matter—both form by crustal extraction. The key here is that shallow mantle depleted domains formed by crustal extraction in the Hadean, so the depleted domain with 142Nd/144Nd that the authors report need not come from the deep mantle (i.e., Bridgmanite).

Line 111. For that reason, we directly measure the 111 Sm and Nd partition coefficients between bridgmanite and melt by performing experiments 112 using the laser-heated diamond anvil cell at pressures between 53 to 97 GPa (see details in the Supplementary material), yielding Dliq-brid(Nd) = 0.24 Å}0.12 and Dliq-brid(Sm) = 0.45 Å}0.10 114 across deep mantle conditions.
Comment: Please discuss how your newly measured partition coefficients compare to prior partition coefficients for Dliq-brid(Nd) and Dliq-brid(Sm)

Line 128. It should be 128 noted that previously published Sm and Nd partition coefficients between bridgmanite and 129 liquid27 are much lower, and their use would result in much lower Nd contents in the solid 130 phase.
Comment: The authors cite the Corgne et al dataset here. What about the Hirose et al. data?
Hirose, K., N. Shimizu, W. van Westrenen, and Y. Fei (2004), Trace element partitioning in Earth’s lower mantle and implications for geochemical consequences of partial melting at
the core-mantle boundary, Phys. Earth Planet. Inter., 146, 249–260, doi:10.1016/j.pepi.2002.11.001.

Line 132. We therefore propose that most 132 of the deep mantle that crystallised from the magma ocean was dominated by bridgmanite and 133 had a high Sm/Nd ratio. This makes it an ideal candidate for producing mantle material with 134 positive 142Nd anomalies.
Comment: This is fine (i.e., a Bridgmanite domain with high Sm/Nd is certainly possible), but I don’t think the authors have ruled out a geochemically depleted Hadean domain that formed in the shallow mantle via crustal extraction. See comments

above.

Line 140. Here, we choose the giant impact that formed the Moon as the 140 last event that melted most of the terrestrial mantle30,31 to generate the last major magma ocean. 141 This event would have erased any previous heterogeneities32, and its timing is unclear though 142 it is estimated to have occurred within the first few hundred million years of Earth's history.
143 Here, we have chosen an age of 4.46 Ga33,34 as suggested by the age of the oldest lunar material 144 but, we have allowed for significant uncertainties by applying an error bar of 50 Ma.
Comment: One problem with this model is the demonstrated survival of 129Xe/130Xe isotope anomalies in the modern mantle. 129Xe/130Xe anomalies had to form in the first 90 million years after accretion (i.e., before 4.477 Ga), during the lifetime of 129I. If all early-formed heterogeneities were erased by the giant impact, then 129Xe/130Xe constrains the timing of the giant impact to have occurred before 4.477 Ga (i.e., before 129I became effectively extinct), to provide time for 129Xe/130Xe anomalies to “grown in” after the giant impact.

Line 151. All three instantaneous solids have higher 151 Sm/Nd ratios than the BSE.
Comment: This does not appear to be true. The Sm/Nd of instantaneous solids during Bridg.+ Fp + Ca-Pv crystallization are all lower than BSE. Please see Fig 2.

Line 157. Due to the very large volume that bridgmanite-rich material represented in the Hadean 157 deep mantle, we suggest that nuggets of this material could have been preserved in a convecting 158 ordinary mantle, contributing to the Fani Maor. source alongside the present-day ordinary 159 mantle (μ142Nd = 0 and ε143Nd = +3.07, assuming a non-chondritic silicate mantle26).
Comment: Why couldn’t nuggets of depleted Hadean upper mantle material—like that identified in superchondritic 176Hf from Hadean zircons—have contributed to Fani Maore?

Line 38. This is also consistent with the Sr and Pb isotopic compositions of the lavas38
Comment: This depends on the 143Nd/144Nd, 87Sr/86Sr and Pb isotopic composition that you select for the sediments. Which values did you select?

Line 177. In that case, the proportion of Hadean bridgmanite would be much higher
177 (>30 %), which makes it difficult to envision how such large quantities could be present in the 178 mantle today.
Comment: It doesn’t need to be present everywhere in the mantle at 30%, just locally in the “nugget” that was entrained from the deep mantle and incorporated into the plume.

Line 198. The presence of such material in their 198 source could potentially explain the rare negative 142Nd anomalies observed in MORBs41 or 199 OIBs42Therefore, both
Comment. Separate sentences with a period.

Supplementary Data 1D. Why are some of the rows colored orange?

Line 66. Time drift was corrected using 11-cycles interpolations.
Comment: Please explain what time drift correction is.

Line 67. Data were systematically corrected8 for 67 Sm and Ce isobaric interferences in case 147Sm/144Nd was higher than 7Å~10-7 or 140Ce/144Nd was 68 higher than 9Å~10-6.
Comment: Why do you use a threshold for application of 140Ce/144Nd? This is not standard? Unusual. For example, why don’t correct for 142Ce when 140Ce/144Nd is 8x10^-6? I don’t see a justification for this.

Line 70. Six measurements were corrected for ≈0.5 ppm Sm 70 contributions on the .^{4}.Nd/.^{44}Nd ratio and DR1402 for a 2.0 ppm contribution. Similarly, Ce 71 interference was negligible in 34 measurements, with only three requiring corrections, with 72 contributions of 1.6 ppm (DR140401) and 6-8 ppm (both measurements of DR070202) on 73 .^{4}.Nd/.^{44}Nd.
Comment: Please make notes of these 9 samples in the tables where the reader can more easily evaluate.

Line 79. typical reproducibility between 1.8 and 3.9 ppm for 142Nd/144Nd
Comment: 1 sigma or 2? RSD or RSE?

Lin3 82. Since JNdi-1 was measured the largest number of measurements in the same 82 conditions, we consider its 2 sd as representative of the external error for all samples (3.1 for
83 142Nd/144Nd).
Comment: What is the 2SD for 142Nd/144Nd of the Ames analyses. Please report here for consistency.

Line 84. Three samples were measured during session 1 and were normalized to JNdi-1 after 84 being corrected using 142Nd/144NdAMES,session 1 / 142Nd/144NdAMES,session 2.
Comment: Two points:
1. I don’t know what is going on here, but it does not have a good appearance. All analyses need to be treated and normalized in exactly the same fashion. I suggest removing the data instead of making a special pleading argument here.
2. You explain how 3 samples were normalized to the standard analyses. How were the others normalized? Important to provide an explicit comparison here.

Line 58. About 800 ng of Nd per analysis was loaded onto zone refined 99.999% Re filaments used in a double filament configuration.
Comment: Folks at Carnegie have indicated that large loads can yield anomalously high 142Nd/144Nd. The mechanism for this is not clear—likely owing to fractionation on the filament--but the result is that Nd load sizes must be incredibly consistent from sample to sample (and consistent between samples and standards).

Line 102. We monitor these Nd ratios for variations exceeding the JNdi-1 102 reproducibility, indicating possible and associated ppm-level μ.^{4}.Nd deviations. DR1802-1 has 103 148Nd and 150Nd excesses (15.6 and 40.4 ppm) and a 145Nd deficit (2.2 ppm – Table S1), mimicking 104 NFS-induced variations (in red in Fig. S2B and Table S1). This suggests that its measured positive 105 μ.^{4}.Nd might be underestimated and should be even more positive.
Comment: It's important for the reader to evaluate this. Please provide plots of μ142Nd vs μ145Nd, μ142Nd vs 148Nd, μ142Nd vs μ150Nd. Plot three different data groups in the plots: JNdi analyzed during the study, all Fani Maoré analyzed during the study, and all Mayotte analyzed during the study. Please show 2SE error bars on all the analyses. This will help evaluate fractionation influences are at work.

Line 58. High-precision Nd isotope analyses were performed on a Nu Instruments TIMS at IPGP
58 following a 5-line acquisition method8.
Comment: Please provide a visual in the paper that shows the cup used for each step for the 5 steps.

Line 211. Incorporation of <0.5% of such recycled sediment (GLOSS-II) is 211 sufficient for its low ε143Nd to balance the high ε143Nd of Hadean bridgmanite and reproduce the value measured for Fani Maore. lavas, while not changing the 212 proportions of Hadean bridgmanite 213 and modern mantle components.
Comment: An issue here is that the GLOSSII 143Nd/144Nd ratio is not known.

Referee #2

(Remarks to the Author)
Review of C. Israel et al., for Nature
"Hadean bridgmanite in the source of a present-day ocean island"
This study reports positive 142Nd anomalies (3.6 to 5.5 parts-per-million (ppm) above the Nd standard) in several volcanic rock samples from the Comoros Archipelago. Because such 142Nd anomalies cannot form after the extinction of 146Sm (half-life: 92 Ma), their presence indicates the incorporation of silicate differentiation products in the mantle source of the Comoros Archipelago formed within the first 500 Ma of Solar System history.
The early history of Earth is one of the most fascinating—yet least constrained—topics that isotope geochemistry can help illuminate. The manuscript's introduction is well written and clearly presents both the hypothesis and the motivation for this study. The paper also reports a substantial dataset from challenging and technically demanding analyses. However, I do not see evidence that the analytical methods represent an improvement over previous studies (e.g., Hyung et al., 2020; Horan et al., 2018; Peters et al., 2018), and I find that the authors' conclusion is not necessarily justified by the data presented. Looking at the supplementary Table SD1A, the samples that were identified having 142Nd anomalies are reported to have 2SE errors of 1–2 ppm, allowing them to be distinguished from the JNdi-1 standard. However, these samples were measured only once. Other samples where repeated measurements were performed have 2SD errors that are on average ~4.2 ppm. This suggests that the 2SE errors of 1–2 ppm of samples measured once are underestimated, and that the 142Nd anomalies would not be significantly different from the terrestrial standard's long-term reproducibility of ±3 ppm. It is therefore unclear how this study overcomes the analytical limitations noted in earlier work. The reported two-year long-term reproducibility of 3.1 ppb for the 142Nd/144Nd ratio in the reference material appears similar to previous published studies. Even if there was 142Nd variability in the samples studied (but the 1–2 ppm resolution would have to be tested by repeated measurements of samples), the data do not seem to require the authors' preferred interpretation—that remnants of a bridgmanite reservoir exist. Alternative explanations, such as recycling of early Hadean crust into the mantle (e.g., Tusch et al., 2022), could be equally plausible. Consequently, I am not convinced that this paper represents a significant advance in understanding that will influence thinking in the field, and would consider publication in another less-selective journal that allows for a longer format.
Line-by-line comments:
• Line 42: heterogeneities of the Hf-W and I-Xe short-lived chronometer can be explained by events after the 50-100 Ma of the Earth (e.g., recycling Hadean crust into the mantle or core-mantle interactions) and thus should be rephrased for clarity.
• Line 50: Many Archean samples display 142Nd variability, but not many locations with those Archean samples. Text should be rephrased for clarity.
• Line 223: Correct the citation.
• Supplementary material: In the first analytical session where the Nd isotope standard AMES #13–#17 was measured, several values in Table SD1B appear anomalous. Some multidynamic and dynamic ratios—particularly the stable 145Nd/144Nd ratio—are off. Also, the 142Nd/144Nd "Static 1" ratios are also off (1.1417 instead of ~1.1418). If any samples were measured during this session and normalized to these standards, the authors should re-examine those results.

Version 1:

Reviewer comments:

Referee #1

(Remarks to the Author)
The authors have done a really good job convincing me that there is a 142Nd anomaly in their data. The time series 142Nd plots and the plots showing the XXXNd vs 142Nd plots show that there is excess 142Nd in their rocks compared to the standard. This does not appear to be caused by fractionation.
However, there are two remaining items that need to be addressed, one easy and the other more difficult:
1. To ensure there is no contribution from 142Ce on the 142Nd, the authors should include a figure of 140Ce/144Nd vs 142Nd/144Nd and put this plot in the supplement. This should be an easy 10-minute job.
2. The issue of the origin of the 142Nd excess in their lavas is a bit more complicated. The authors argue that the 142Nd excess is due to bridgmanite, but reviewer #2 and I both agree that this is not a unique explanation for the excess 142Nd. This is the only remaining issue that lingers. I appreciate that the authors have augmented their discussion to address this issue.
A. The authors write: “mantle depletion is only firmly recorded [in zircons] after ~3.8 Ga”. Comment: The zircons record enriched mantle reservoirs prior to 3.8 Ga. Mass balance requires there were complementary depleted reservoirs. I’m not sure how the zircon record excludes the presence of depleted reservoirs prior to 3.8 Ga.
B. The authors write: “While the survival of localised Hadean depleted domains cannot be ruled out, the constraints provided independently by geophysical and geodynamical studies27 focusing on mixing time in the mantle, make this option a low-probability scenario.” Comment: I’m not sure the studies cited provide probabilities of one scenario occurring vs the other scenario occurring. The probability assigned is the authors’ perspective, but not really evaluated in the studies cited.
C. To frame this a bit differently, the authors would need to exclude the presence of Hadean depleted reservoirs before they can convince the community that bridgmanite is the only remaining viable explanation for the 142Nd in their lavas. I don’t think this can be done with existing evidence, but I would be very happy if the authors could find such evidence. Alternatively, the authors can simply acknowledge that bridgmanite is simply one possible explanation, but survival of early depleted mantle domains cannot be excluded. Upper mantle material is sent to the lower mantle today (depleted mantle lithosphere attached to slabs, etc), and upper mantle communication with lower mantle was not unlikely in the Hadean. Upper mantle sent into the lower mantle can be preserved for long time periods due to slower convective motions in the lower mantle. I have no skin in this game—I would be perfectly happy to be convinced that bridgmanite is the only explanation here—but I am not yet convinced that bridgmanite is the only explanation. The authors either need to acknowledge their (very interesting) data do not have a unique explanation in this regard, or they need to convince me and the other reviewer that bridgmanite is the only explanation. In either case, I would be supportive of publication.

Referee #2

(Remarks to the Author)
The revised manuscript is well written, and the newly added discussion on early crust extraction, as well as the updates to the supplementary materials, represent meaningful improvements. I also acknowledge the substantial amount of work that has gone into this study. However, as Carl Sagan famously remarked, “extraordinary claims require extraordinary evidence,” and the data presented here are not as unequivocal as the authors suggest.
Regarding the significance of the reported 142Nd anomalies in Fani Maoré lavas: in their response, the authors state that “unfortunately, we do not really see what could be changed in the manuscript or the supplementary material to convince the reviewer of the existence of the positive anomalies.” However, it is not only me who needs to be convinced—any claim of statistically significant 142Nd deviations must be rigorously supported for the broader community. As currently written, the manuscript makes statements (e.g., “the significant 142Nd excess in Fani Maoré lavas…” in line 73, or line 190) that require quantitative justification.
If the co-authors wish to assert that these anomalies are significant, this must be demonstrated using appropriate statistical approaches. For example, a test comparing the sample values with the long-term JNdi-1 average (given the reported reproducibility of 3.1 ppm) could be used to show that the probability of the observed offsets occurring by chance is <5% (i.e., $p<0.05$). Without such analysis, these statements are not yet supported.
Also, line 63 states “This enhanced precision overcomes the analytical limitations of previous studies…”. As noted in my previous review, the precision reported here does not exceed that of several published works. For example, both Saji et al. (2016) and Wang and Carlson (2022) reported ~ 2.5 ppm precision for 142Nd/144Nd ratios on the JNdi-1. Therefore, previous publications seem to report a higher precision compared to this study. Line 63 should be revised to accurately describe the analytical capabilities of this study.
Given these unresolved issues, I remain unconvinced that the present study meets the evidentiary threshold required for publication in Nature.

Version 2:

Reviewer comments:

Referee #1

(Remarks to the Author)
Summary:

The authors have done a thorough job or reframing the arguments in the manuscript to better reflect uncertainties in how 142Nd anomalies are preserved in the modern convecting mantle.
I actually enjoyed reading the revised arguments, particularly the new argument acknowledging that the 142Nd anomaly can be generated in the upper mantle--and such an anomaly could explain the anomalous 142Nd in OIB--but they use a strong geodynamic argument to explain why an upper mantle origin is unlikely.

Additionally, I would like to point out that they have convincingly shown that they can resolve small 142Nd anomalies in OIB. Their long-term precision is really extraordinary, but they have made a series of diagrams (Figs S2, S3, and S4) that go a long way toward convincing me that the 142Nd anomalies are real. The instrument (Nu TIMS) uses fully modern electronics (the electronics in the Thermo Tritons are >30 years old!) and I believe this allows them to push the frontiers of high-precision analysis.

Below I provide very minor comments that request a bit more clarity in the authors' language. Otherwise, this manuscript is ready for prime time. I hope that it can be published without delay.

Specific comments:
1. The authors wrote: The survival of localised Hadean 339 depleted domains originating from the upper mantle thus cannot be ruled out but meeting the 340 conditions for it to have survived up to today in significant amounts makes this option a low341 probability scenario.
Comment: As requested in my prior review, the authors go through the exercise of showing that depleted upper mantle domains can generate the 142Nd anomalies that they observe in OIB. Critically, they go one step further and show, using geodynamic arguments, that it is difficult to preserve a 142Nd anomaly in an upper mantle reservoir, making its preservation a low probability event. This is an ideal response, and makes their preferred interpretation--142Nd anomalies are generated by bridgmanite formation from a magma ocean--a more realistic model.

2. Sizes of reservoirs. The following 4 comments are related.
A. The authors wrote: To match 89 the µ142Nd of +3.2, and the e143Nd measured at Fani Maor., requires ~90% of this material in 90 the source (see Supplementary Material for details), a volume so significant that it raises serious 91 questions about its preservation30 within the convecting mantle for ~4.3 billion years.
Comment: I'm not sure I follow what "material in the source" refers to. Are the authors suggesting that 90% of the entire mantle is bridgmanite, or 90% of the plume? If 90% of the mantle is bridgmanite with the µ142Nd of +6, then it's easy to preserve the +6 anomaly for 142Nd--most of the mantle is made of this material--so no serious questions are raised.
B. The authors wrote: Mitigating this requires identifying a process that generates a reservoir which is (1) large 93 enough for a small fraction of it to realistically be preserved,
Comment: Again, I'm not sure I follow. The authors seem to suggest that it is difficult to find a process that preserves a small part of a very large reservoir with positive 142Nd. I'm not sure I see the problem here. Larger reservoirs are easy to preserve than smaller reservoirs.
C. The authors wrote: has a much stronger 94 positive 142Nd anomaly so as to necessitate minute quantities to produce the observed effects.
Comment: Why does the reservoir with positive 142Nd have to be smaller (in mass) and have larger 142Nd? Why is it necessary (the authors write necessitate) that the positive 142Nd reservoir is sampled in minute quantities?
D. The authors wrote: For a depletion age of 320 4.46 Ga, the depleted reservoir would have a µ142Nd of +19 and for a depletion age of 4.3 Ga it would be +6 (Fig. S9). Using these values and the Nd concentration of 321 DMM46, we calculate that 322 about 26% of such material is required to reproduce Fani Maor. µ142Nd if the depletion occurred 323 at 4.46 Ga. Any later formation age for the depleted reservoir involves higher proportions in the 324 source of Fani Maor., up to ≈70% for an age of 4.3 Ga.
Comment: The amount of material required to reproduce Fani Maore depends on the µ142Nd (µ142Nd = +19 vs µ142Nd = +6). Each unique µ142Nd will require a unique percentage of material. Provide both in a clear way.
E. In that case, the proportion of Hadean bridgmanite would 180 be much higher (>30 %), making it 181 difficult to envisage how such large quantities could be present in the mantle today.
Comment: This seems to be a different version of earlier arguments about reservoir size. In this case, the argument is that the authors question how a relatively large reservoir could be preserved over 4.5 Gyr. The issue is that this is framed as an opinion, without supporting evidence. If it is difficult to preserve this mass of Bridgmanite for 4.5Ga, explain why and provide citations. This will make the argument stronger.
F. The shallow model 188 requires the source to contain 28–90% of Hadean material, whereas the deep model only 189 requires 9–11%.
Comment: See previous comment ("comment E"). Whether a reservoir or one size (28-90%) or another (9-11%) can be preserved is framed as an opinion, so this is not a strong argument. In the supplement, the authors use a logical and convincing geodynamic argument to deal preservation of the former (upper mantle) reservoir, where they write: "For Fani 335 Maore's source to be largely composed of such depleted mantle requires that the Hadean 336 component is large enough to be preserved unaltered for more than 4 Gyr. As it formed in the 337 upper mantle, it needs to be dense enough to sink at the bottom of the lower mantle where it could 338 potentially escape the efficient stirring due to convection." I strongly suggest that the authors use the same argument here."

Open Access This Peer Review File is licensed under a Creative Commons Attribution 4.0 International License, which permits use, sharing, adaptation, distribution and reproduction in any medium or format, as long as you give appropriate credit to the original author(s) and the source, provide a link to the Creative Commons license, and indicate if changes were made.
In cases where reviewers are anonymous, credit should be given to 'Anonymous Referee' and the source.
The images or other third party material in this Peer Review File are included in the article’s Creative Commons license, unless indicated otherwise in a credit line to the material. If material is not included in the article’s Creative Commons license and your intended use is not permitted by statutory regulation or exceeds the permitted use, you will need to obtain permission directly from the copyright holder.
To view a copy of this license, visit https://creativecommons.org/licenses/by/4.0/

Manuscript Number 2025-06-16053

We would like to thank the editor and the two reviewers for the constructive comments they provided. We took them all into account and we believe that the quality of the manuscript has now been improved.

Please find below our answers shown in blue to all comments (shown here in black). Changes made to the manuscript itself are indicated in light blue and a version of the manuscript with highlighted changes is also provided.

Referee #1:

Review of paper entitled “Hadean bridgmanite in the source of a present-day ocean island” by Claudine Israel, Catherine Chauvel, James Badro, Hui Chen, Cecile Hebert, Edward Inglis

The authors provide new high-precision ^{142}Nd data on OIB from the Comoros archipelago. The data are the result of an impressive analytical: The authors report a new level of analytical precision that most prior papers, with the exception of the early work reported by Caro (who reported 1sigma=1ppm precision on JNdi). It is particularly impressive and useful that the authors report new mineral-melt partition coefficients for Sm and Nd for bridgmanite.

There has been a lot of interest in identifying ^{142}Nd anomalies preserved in Phanerozoic lavas, as this would show that the products of Hadean silicate differentiation of the Earth have been preserved in the mantle for 4.5 Gyr. Unfortunately, the size of the anomalies—if they exist—are on the order of the precision that can be obtained with modern mass spectrometry. Thus, it remains unclear whether true ^{142}Nd anomalies have been detected in Phanerozoic lavas. The authors employ a new generation of mass spectrometer (a TIMS manufactured by Nu Instruments) to push the limits of analytical precision a report a new level of precision that, if demonstrated to be correct, makes this a paper that is suitable for publication in Nature.

We would like to thank the reviewer for this positive feedback!

However, there are some key issues that the authors will need to deal with before this manuscript is ready for primetime:

1. To better evaluate (and gain more confidence in) the μ^{142}Nd analyses, it would help to see plots that show whether they correlate with with ^{143}Nd/^{144}Nd, with Pb isotopes, and/or with trace element rations (i.e., La/Sm, Nb/Th, Ce/Pb, Sm/Yb, etc etc)? A suite of plots could go into the supplement and would take the authors perhaps 2 hours to make. If the lavas with anomalous 142Nd are somehow different geochemically, then this would be useful to see.

We would like to thank the reviewer for the suggestion. The requested plots have been added as Supplementary Figure S5 and a new paragraph entitled “Relationship to other radiogenic isotopes and trace elements” has been added in the Supplementary Material lines 128–138.

As can be seen in Figure S5 (also shown below), there is no correlation with any of the ratios, indicating that the ^{142}Nd anomaly is disconnected from these parameters. This indicates that the process responsible for the ^{142}Nd anomaly has no impact on the other variables, which is consistent with the presence of Hadean bridgmanite in the source.

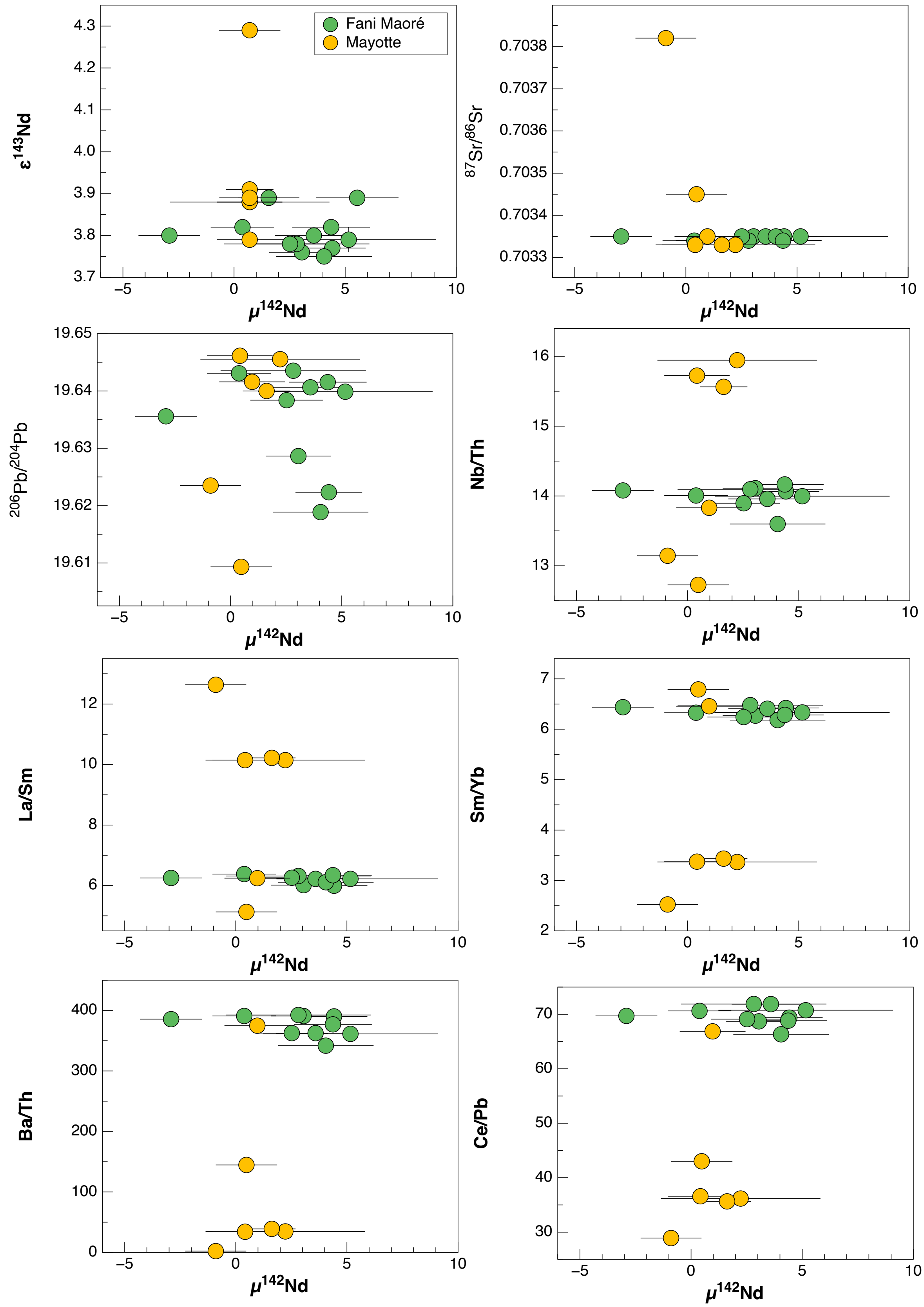

2. The authors attribute the ^{142}Nd to early Sm/Nd fractionation by bridgmanite precipitation from a terrestrial magma ocean. Thus, they invoke a lower mantle process. But I don't understand how the authors rule out depleted domains that formed in the Hadean? These

depleted domains need not be volumetrically large. They just need to have formed early—to generate high $^{142}Nd/^{144}Nd$ — and be preserved. There is ample evidence from the Hadean zircon record for early (Hadean) depleted mantle domains with superchondritic eps^{176}Hf. Perhaps there wasn't a DMM domain exactly as we know it today, but Hadean zircons tell us that there were geochemically depleted mantle domains formed by continental crust extraction. Crust extraction from the upper mantle in the Hadean resulted in depleted domains. Whether you call them DMM or depleted domains doesn't really matter—both form by crustal extraction. The key here is that shallow mantle depleted domains formed by crustal extraction in the Hadean, so the depleted domain with $^{142}Nd/^{144}Nd$ that the authors report need not come from the deep mantle (i.e., Bridgmanite). I don't think the authors have excluded the possibility of an upper mantle origin for high $^{142}Nd/^{144}Nd$ observed in the Comores hotspot lavas.

We agree that Hadean zircons have been identified, essentially detrital zircons. However, their global significance is disputed (see recent review from Vervoort et al., 2025, Annual Rev. of Earth and Plan. Sciences). Very few zircons exhibit positive εHf before 3.8 Ga, and no Eoarchean samples provide evidence of a Hadean depleted source (Whitehouse et al., 2017, Gond. Res.).

While debate is certainly beyond the scope of our paper, we agree that the possibility of a depleted mantle formed by crustal extraction needs to be considered. We evaluate this hypothesis in the section entitled "Origin of positive ^{142}Nd anomalies" in the main manuscript (lines 77-99). This section has been completely rewritten to clarify the message and emphasise the main arguments showing that a depleted mantle formed by crustal extraction is unlikely to have generated the ^{142}Nd anomalies in Fani Maoré lavas.

Two new sections have also been added in the Supplementary material entitled "Isotope modelling: assumptions about the composition of the mantle" and "Modelling a Hadean, depleted mantle by crustal extraction" (lines 140–155 and 157–182) to provide further details.

We hope that the new text properly addresses the comment.

3. It's important for the reader to be able to evaluate fractionation. This is most easily done by providing plots of µ142Nd vs µ145Nd, µ142Nd vs 148Nd, µ142Nd vs µ150Nd. Make 3 panels (µ142Nd vs µ145Nd, µ142Nd vs 148Nd, µ142Nd vs µ150Nd) and plot 4 different data groups in the plots using different symbols: JNdi analyzed during the study, Ames analyzed during the study, all Fani Maoré analyzed during the study, and all Mayotte analyzed during the study. Please show 2SE error bars on all the analyses. This is easy to do—a 30-minute job for the authors--and will help evaluate fractionation influences are at work, thus lending more confidence in their work. Please include these plots in the supplement as other readers will want to see the data presented this way.

These plots are now included in the Supplementary Material as Fig. S3.

The text has been modified accordingly in the Supplementary Material lines 111–113: "In Figure S3, anomalous variations can be identified as they may exceed the typical variability of pure standard solutions and can serve as indicator for potentially biased $^{142}Nd/^{144}Nd$ data."

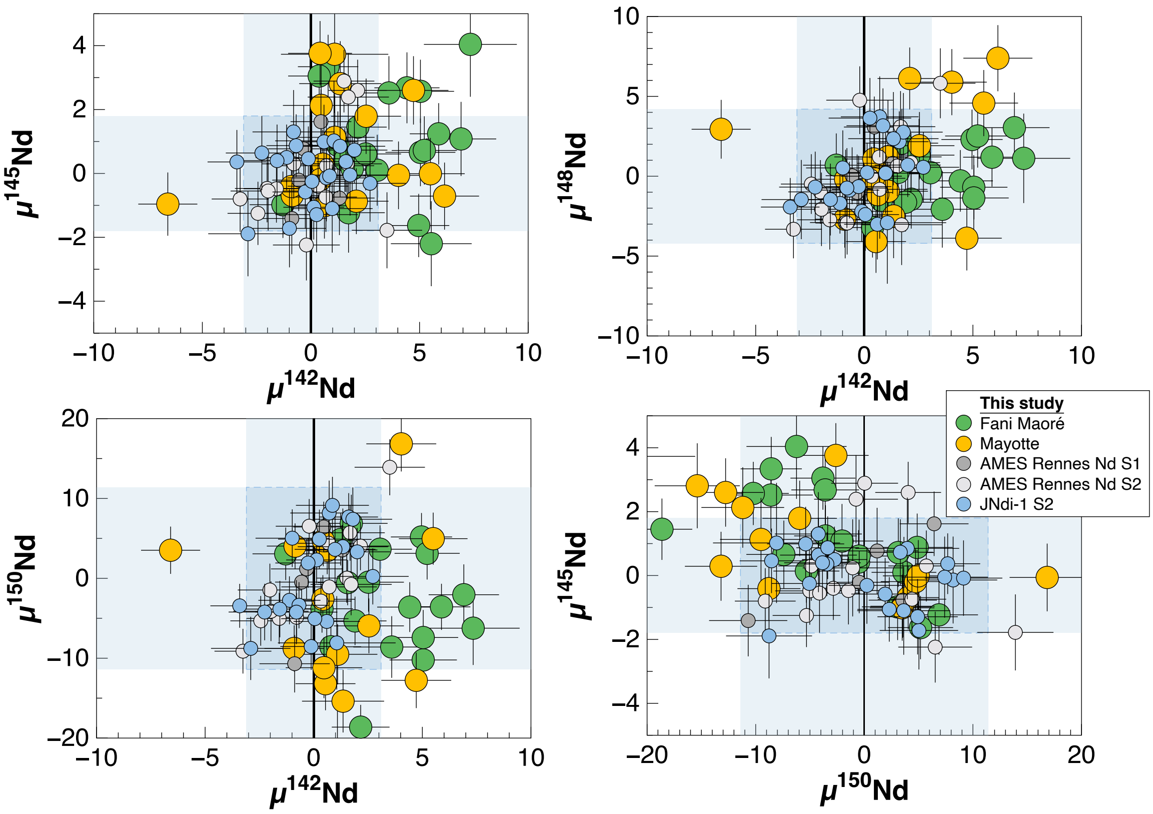

4. It will be important to show all analyses of µ142Nd, µ145Nd, 148Nd, and µ150Nd analyses vs time. Thus, there will be 4 panels (µ142Nd, µ145Nd, 148Nd, and µ150Nd analyses vs time). In each panel show 4 different symbols: JNdi, Ames, and the two different Comoros volcanoes. Please include these plots in the supplement, as other readers will want to see the data presented this way.

These plots have been included in the Supplementary Material as Fig. S2.

The text has been modified accordingly in the Supplementary Material lines 88–89 and 103–104 "Details on the individual measurements of AMES Rennes Nd and JNdi-1 are given in Supplementary Data 1B and are shown in Figures S2 and S3." And "All Nd isotope measurements of samples and pure Nd reference materials acquired during the course of this study are reported in Supplementary Data 1 and Figures S2 and S3."

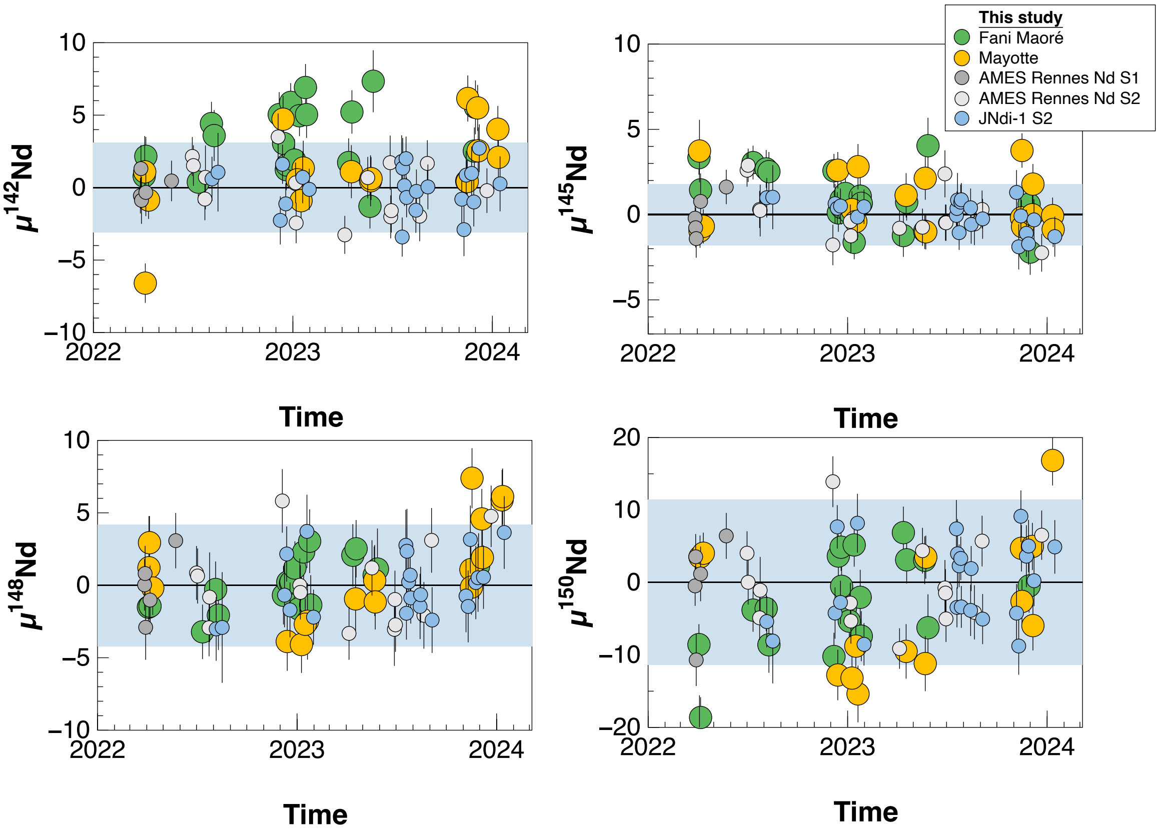

Below I provide additional specific comments that should be useful for the authors as they revise the manuscript:

Line 41. High 3He/4He in Hawaii lavas 41 indicates that their source contains primordial mantle material4.

Comment: Would be worth saying that high "3He/4Hd in OIB", instead of just Hawaii. This is because other OIB—Iceland, Samoa, Galapagos—have 3He/4He that is similar to or higher than Hawaii.

Modified in the main manuscript lines 40-41: "High $^{3}He/^{4}He$ in OIB (e.g., Iceland, Samoa, Galapagos, Hawaii) indicates that their source contains primordial mantle material[4]."

Line 50. Many Archean samples **preserved in continental settings** display excesses and deficits in 142Nd up to 20 parts per million or ppm (see compilation in 10) testifying of the active decay of 146Sm.

Comment: To distinguish from the mantle, and to engage broader leadership, add the suggested text to this sentence.

Modified in the main manuscript lines 51-53: "Some Archean samples preserved in continental settings display excesses and deficits in ^{142}Nd up to 20 parts per million or ppm (see compilation in [10]) testifying of the active decay of ^{146}Sm."

Line 54. Several studies have sought to identify 142Nd isotope variations in modern MORB and OIB.

Comment: This is a great place to cite proposed anomalies measured in phanerozoic lavas, which are cited elsewhere in the paper.

We have added the relevant references to the proposed anomalies.

Modified in the main manuscript lines 55-57: "Several studies have sought to identify ^{142}Nd isotope variations in modern MORB and OIB[11,12,13], but the overwhelming majority of published data are within the typical 5-ppm error of the mantle reference composition (see Fig. 1)."

Line 83. Today, this depleted material would have a µ142Nd of +19 to +4 if formed within the first 100 to 300 million years of Earth's history.

Comment: explain how you arrived at this range in µ142Nd.

This is a very good suggestion. The entire section of the main manuscript entitled "Origin of positive ^{142}Nd anomalies" has been rewritten to take into account these comments.

All key parameters are now provided in Supplementary Table S2 and the calculation procedure is now described in a new section of the Supplementary Material ("Modelling a Hadean, depleted mantle by crustal extraction" (lines 157–181).

Line 84. To reproduce the µ142Nd of +3.2 measured at Fani Maoré would require up to 80% of such Hadean depleted material mixed up with ordinary mantle to form the source of the volcano.

Comment: 80% assumes a particular value in the range of µ142Nd (+19 to +4) that you provided. Which value did you assume? Also, this percentage requires assumptions about the source concentrations of normal mantle and the Hadean depleted material, but you did not provide the concentrations.

We would like to thank the reviewer for highlighting the ambiguity in our sentence. Please note that the entire section of the main manuscript entitled "Origin of positive ^{142}Nd anomalies" has been rewritten to take into account these comments.

Moreover, the calculation procedure as well as all key parameters are now provided in a new section of the Supplementary material entitled "Modelling a Hadean, depleted mantle by crustal extraction" (lines 157–181) and in Supplementary Table S2. We hope that these changes clarify the way calculations were performed.

Line 86. Such scenario seems quite unlikely because it would suggest that a depleted mantle similar to the modern DMM was formed as early as the Hadean.

Comment: There is a logical step missing here. The authors assume that the mixing had to happen in the Hadean, but there is no requirement for this. Mixing between the Hadean depleted material and post-Hadean DMM could occur after the Hadean. For example, why couldn't Hadean depleted material and mix with Proterozoic DMM during the Phanerozoic? Or Hadean depleted material and mix with Phanerozoic DMM during the Phanerozoic? And so forth?

This sentence has been removed, and the entire section of the main manuscript entitled "Origin of positive ^{142}Nd anomalies" has been rephrased to take into account these comments (lines 77–100). We hope that it is now clearer.

Line 86. Such scenario seems quite unlikely because it would suggest that a depleted mantle similar to the modern DMM was formed as early as the Hadean. This implies a very high crustal extraction rate which seems to be difficult to reconcile with current models of crustal growth[16]. An alternative explanation involves the creation, within the Hadean mantle, of enriched and depleted domains producing both positive and negative µ142Nd values with time.

Comment: I don't understand why depleted domains couldn't form in the Hadean? These depleted domains need not be volumetrically large. They just need to have formed early and be preserved. There is ample evidence from the zircon record for early (Hadean) depleted mantle domains with superchondritic eps176Hf. Perhaps there wasn't a DMM domain exactly as we know it today, but Hadean zircons tell us that there were geochemically depleted mantle domains formed by continental crust extraction. Crust extraction in the Hadean resulted in

deplted domains. Whether you call them DMM or depleted domains doesn't really matter—both form by crustal extraction. The key here is that shallow mantle depleted domains formed by crustal extraction in the Hadean, so the depleted domain with 142Nd/144Nd that the authors report need not come from the deep mantle (i.e., Bridgmanite).

This comment repeats what was listed as main point 2 above. We modified the text in the main manuscript as mentioned in the answer to main point 2 (see lines 77–99).

Line 111. For that reason, we directly measure the Sm and Nd partition coefficients between bridgmanite and melt by performing experiments using the laser-heated diamond anvil cell at pressures between 53 to 97 GPa (see details in the Supplementary material), yielding Dliq-brid(Nd) = 0.24 ±0.12 and Dliq-brid(Sm) = 0.45 ±0.10 across deep mantle conditions.

Comment: Please discuss how your newly measured partition coefficients compare to prior partition coefficients for Dliq-brid(Nd) and Dliq-brid(Sm)

We added the paragraph copied below in the Supplementary Material to give the readers a little bit of background on the reasons why our partition coefficients differ from those of Corgne.

Modified in the Supplementary Material lines 217–227: "Our data can be compared to the Corgne et al.[31] data which are multi-anvil experiments that have demonstrably achieved equilibrium (long-duration experiments, in contrast to the shorter runs reported elsewhere). In addition, the lithology investigated by Corgne et al. closely matches ours, and by extension the bulk silicate Earth (a pyrolite composition doped with trace elements). Our partition coefficients for Sm and Nd are an order of magnitude higher than those measured at 25 GPa and 2300 °C. This can be expected for multiple reasons, from thermodynamics and crystal chemistry. Our experiment is at 65 GPa and 4000 °C. At higher temperature (a natural consequence of increasing liquidus temperature with pressure), element partitioning tends to unity (equipartition principle of statistical physics). At higher pressure, the difference in compressibility and size of the host site for large cations (between crystal and melt) drops as expected from lattice strain theory, so that partitioning should also tend towards unity."

Line 128. It should be noted that previously published Sm and Nd partition coefficients between bridgmanite and liquid[27] are much lower, and their use would result in much lower Nd contents in the solid phase.

Comment: The authors cite the Corgne et al dataset here. What about the Hirose et al. data?

Hirose, K., N. Shimizu, W. van Westrenen, and Y. Fei (2004), Trace element partitioning in Earth's lower mantle and implications for geochemical consequences of partial melting at the core-mantle boundary, Phys. Earth Planet. Inter., 146, 249–260, doi:10.1016/j.pepi.2002.11.001.

This is an interesting point! We have partially addressed this issue in the comment above.

We chose to cite and use the Corgne et al. (2005, GCA) data because it represents the only multi-anvil experimental study that has demonstrably achieved equilibrium (long-duration experiments, in contrast to the shorter runs reported elsewhere, including but not limited to Hirose, 2004. In addition, the lithology investigated by Corgne et al. closely matches ours, and by extension the bulk silicate Earth (a pyrolite composition doped with trace elements). In comparison, Hirose (2004, Phys. of the Earth and Plan. Int.) employed either MORB or a KLB-1 peridotite composition modified with 10% $CaSiO_3$. Since $CaSiO_3$ is the principal host phase for LILE/REE in the deep mantle, these experiments are not directly applicable to geochemical modelling of the BSE (and cannot be directly and reliably compared with ours). For this reason, the partition coefficients reported by Corgne et al. constitute the most comprehensive and reliable dataset at 25 GPa, and it is therefore unsurprising that they have become the preferred choice in more recent studies.

Modified in the note of Table S4 "Note: Previously published partition coefficients for Sm and Nd in bridgmanite in shallow lower mantle conditions were 0.053 and 0.02 in Corgne et al.[31].".

Line 132. We therefore propose that most of the deep mantle that crystallised from the magma ocean was dominated by bridgmanite and had a high Sm/Nd ratio. This makes it an ideal candidate for producing mantle material with positive 142Nd anomalies.

Comment: This is fine (i.e., a Bridgmanite domain with high Sm/Nd is certainly possible), but I don't think the authors have ruled out a geochemically depleted Hadean domain that formed in the shallow mantle via crustal extraction. See comments above.

This comment has already been answered above (main point 2) in this rebuttal letter.

Line 140. Here, we choose the giant impact that formed the Moon as the last event that melted most of the terrestrial mantle[30,31] to generate the last major magma ocean. This event would have erased any previous heterogeneities[32], and its timing is unclear though it is estimated to have occurred within the first few hundred million years of Earth's history.

Here, we have chosen an age of 4.46 Ga[33,34] as suggested by the age of the oldest lunar material but, we have allowed for significant uncertainties by applying an error bar of 50 Ma.

Comment: One problem with this model is the demonstrated survival of 129Xe/130Xe isotope anomalies in the modern mantle. 129Xe/130Xe anomalies had to form in the first 90 million years after accretion (i.e., before 4.477 Ga), during the lifetime of 129I. If all early-formed heterogeneities were erased by the giant impact, then 129Xe/130Xe constrains the timing of the giant impact to have occurred before 4.477 Ga (i.e., before 129I became effectively extinct), to provide time for 129Xe/130Xe anomalies to "grown in" after the giant impact.

This is an interesting point! The survival of ^{129}Xe anomalies in the modern mantle is indeed a crucial topic, though it is much debated, and it presents a challenge to simple models of mantle homogenisation:

- Recent studies have shown that mantle melting does not necessarily erase noble gas signatures. For example, Parai et al. (2021, GCA) demonstrated that anomalies can persist in deep, isolated reservoirs even after large-scale melting.
- Anomalies of ^{129}Xe formed within the first 90 Ma are compatible with our model, particularly in light of the recently refined age of the Moon of 4.502 ± 0.020 Ga (Schneider and Kleine, 2025, EPSL), which falls within our adopted range of 4.46 ± 0.05 Ga.

The formation of ^{129}Xe anomalies after the Giant Impact can therefore not be ruled out.

In addition, Schneider and Kleine (2025) recently published a new constraint for the age of the giant impact, which we now refer to on line 149 of the main manuscript.

Modified in the main manuscript lines 148–150: "This event would have erased any previous heterogeneities[40], and its timing is unclear though it is estimated to have occurred either within the first 65 million years of Earth's history[41] or up to 80 millions years later[42]."

Line 151. All three instantaneous solids have higher Sm/Nd ratios than the BSE.

Comment: This does not appear to be true. The Sm/Nd of instantaneous solids during Bridg.+ Fp + Ca-Pv crystallization are all lower than BSE. Please see Fig 2.

This comment is extremely useful to us! We realize the issue arose from a lack of clarity in our manuscript. The reviewer interpreted the "three stages" as referring to the three mineral phases (Brg, Fp, and CaPv). What we intended, however, was to describe three distinct stages of magma ocean crystallization: 10%, 35%, and 75%. At these respective stages, the crystallizing phases are Brg alone (10%), and Brg + Fp (35% and 75%). CaPv does not crystallize until ~90% solid fraction (i.e., 10% residual melt remaining).

Modified in the main manuscript lines 153–159: "We chose three snapshots during magma ocean solidification (10%, 35% and 75% of crystallisation – see Fig. 3) to investigate the isotopic evolution of the solid phases. These correspond to a magma ocean that has solidified 10% of its mass and only crystallised bridgmanite, and then 35 and 75% of its mass, crystallising a mixture of bridgmanite and ferropericlase. In all these cases, the Sm/Nd ratio of the instantaneous solid is significantly higher than that of both the BSE or a mantle depleted by crust extraction."

We believe this change in the wording makes it clear that none of the three steps involves CaPv. We are grateful to the reviewer for highlighting this point of confusion.

Line 157. Due to the very large volume that bridgmanite-rich material represented in the Hadean deep mantle, we suggest that nuggets of this material could have been preserved in a convecting ordinary mantle, contributing to the Fani Maoré source alongside the present-day ordinary mantle (μ^{142}Nd = 0 and ε^{143}Nd = +3.07, assuming a non-chondritic silicate mantle[26]).

Comment: Why couldn't nuggets of depleted Hadean upper mantle material—like that identified in superchondritic ^{176}Hf from Hadean zircons—have contributed to Fani Maore?

This comment relates to the main point 2 and was answered earlier in the rebuttal letter.

Line 38. This is also consistent with the Sr and Pb isotopic compositions of the lavas

Comment: This depends on the ^{143}Nd/^{144}Nd, ^{87}Sr/^{86}Sr and Pb isotopic composition that you select for the sediments. Which values did you select?

All recycling model calculations are based on the present-day isotope composition and parent/daughter ratio for GLOSS-II as reported in Plank, 2014, ToG. The output isotopic composition result from the recycling model as described in Chauvel et al., 2008 for a recycling age of 2 Ga.

Details are provided in the Supplementary Material lines 274–278 "Here we use GLOSS-II (Global Subducted Sediments II), the average composition of subducted sediments suggested by Plank[38] and assume that the recycled sedimentary material has an age of 2 Ga, which implies no m^{142}Nd anomaly. We calculate its present-day Nd isotopic evolution (Table S3) using its published ^{143}Nd/^{144}Nd and Sm/Nd ratios[38] (Table S2) and the recycling model calculations of Chauvel et al. [35] as illustrated in Figure S6." and in the Table caption lines 445–446 "The Nd and Sm content and ^{143}Nd/^{144}Nd ratio of GLOSS-II (Global Oceanic Subducted Sediments) are modelled compositions of average subducted sediment[32]".

For further clarification, we also added a schematic (Fig. S5) that illustrates the recycling model calculations.

Line 177. In that case, the proportion of Hadean bridgmanite would be much higher (>30 %), which makes it difficult to envision how such large quantities could be present in the mantle today.

Comment: It doesn't need to be present everywhere in the mantle at 30%, just locally in the "nugget" that was entrained from the deep mantle and incorporated into the plume.

We are grateful for this suggestion. Nevertheless, it is necessarily more difficult to preserve a large volume of ancient material than a small volume, due to the efficiency of convection (e.g., Samuel et al., 2011, GRL, Tackley et al., 2007, ToG; Van Keken et al., 2013, Physics and Chemistry of the Deep Earth).

Modified in the main manuscript lines 96-98: "While the survival of localised Hadean depleted domains cannot be ruled out, the constraints provided independently by geophysical and geodynamical studies[27] focussing of mixing time in the mantle, make this option a low-probability scenario."

Line 198. The presence of such material in their source could potentially explain the rare negative ^{142}Nd anomalies observed in MORBs[41] or OIBs[42]Therefore, both

Comment. Separate sentences with a period.

Modified in the main manuscript line 206.

Supplementary Data 1D. Why are some of the rows colored orange?

These orange rows were supposed to identify the samples with slightly positive or negative $\mu^{145,148,150}$Nd values. As they seem to be confusing, these colours are no longer showed.

Line 66. Time drift was corrected using 11-cycles interpolations.

Comment: Please explain what time drift correction is.

The time-drift correction is explained thoroughly in Israel et al., 2025, Chem. Geol. and we feel that it should not be repeated here.

Modified in the Supplementary Material lines 57-58: "High-precision Nd isotope analyses were performed on a Nu Instruments TIMS at IPGP following a 5-line acquisition method thoroughly detailed in Israel et al.[8]".

Line 67. Data were systematically corrected[8] for Sm and Ce isobaric interferences in case 147Sm/144Nd was higher than 7×10^{-7} or ^{140}Ce/^{144}Nd was higher than 9×10^{-6}.

Comment: Why do you use a threshold for application of 140Ce/144Nd? This is not standard? Unusual. For example, why don't correct for 142Ce when 140Ce/144Nd is 8x10^-6? I don't see a justification for this.

We chose to apply a threshold for Ce interference correction to prevent the introduction of additional analytical error from overcorrecting. As detailed by Israel et al. (2025, Chem. Geol.), a ^{140}Ce/^{144}Nd ratio of 9×10^{-6} produces a bias of approximately 1 ppm in the ^{142}Nd/^{144}Nd ratio. This bias is roughly equivalent to the typical two-standard error margin of error on a single measurement. Based on the average background ratio in pure Nd standards ($3\times10^{-6}\pm3$) for analyses at ≈5-6 V of $^{142}Nd^{+}$, our detection limit for a true Ce signal is approximately 5×10^{-6} for ^{140}Ce/^{144}Nd. Ratios at or below this limit are difficult to distinguish from background noise. Applying a correction in these cases could introduce more error than it removes. Therefore, the 9×10^{-6} threshold serves as a robust compromise, ensuring that we only correct for statistically significant interferences without the risk of overcorrection.

We now provide more context in the supplementary material.

Modified in the Supplementary Material lines 68–73: "Data were systematically corrected[8] for Sm and Ce isobaric interferences in case ^{147}Sm/^{144}Nd was higher than 7×10^{-7} or ^{140}Ce/^{144}Nd was higher than 9×10^{-6}. This allows reliable correction and prevents overcorrection that might increase errors. Detection threshold to consistently distinguish Sm and Ce signal from the background noise during our analyses is established at ^{147}Sm/^{144}Nd > 5×10^{-7} and ^{140}Ce/^{144}Nd > 5×10^{-6} (for I$^{142}Nd^{+}$ ~5 V – see Figure 9a,c in [8])."

Line 70. Six measurements were corrected for ≈0.5 ppm Sm contributions on the ^{142}Nd/^{144}Nd ratio and DR1402 for a 2.0 ppm contribution. Similarly, Ce interference was negligible in 34 measurements, with only three requiring corrections, with contributions of 1.6 ppm (DR140401) and 6-8 ppm (both measurements of DR070202) on ^{142}Nd/^{144}Nd.

Comment: Please make notes of these 9 samples in the tables where the reader can more easily evaluate.

We would like to thank the reviewer to point that out. The Sm and Ce corrections are now annotated in Supplementary Data 1A.

Line 79. typical reproducibility between 1.8 and 3.9 ppm for 142Nd/144Nd

Comment: 1 sigma or 2? RSD or RSE?

As mentioned in the Supplementary Material line 84, the reproducibility is reported as 2 sd.

Line 82. Since JNdi-1 was measured the largest number of measurements in the same conditions, we consider its 2 sd as representative of the external error for all samples (3.1 for $^{142}Nd/^{144}Nd$).

Comment: What is the 2SD for 142Nd/144Nd of the Ames analyses. Please report here for consistency.

The reproducibility on both AMES Rennes Nd and JNdi-1 is reported as 2 sd. In the text, we focus on the reproducibility of JNdi-1 because it is the main standard used in literature.

Modified in the Supplementary Material lines 83–87: "They gave similar isotope ratios within error, with typical reproducibility (2 sd) between 1.8 and 3.9 ppm for $^{142}Nd/^{144}Nd$, 2.0 to 3.8 ppm for $^{143}Nd/^{144}Nd$, 1.8 to 3.1 ppm for $^{145}Nd/^{144}Nd$, 4.2 to 5.6 ppm for $^{148}Nd/^{144}Nd$ and 11.4 to 11.9 for $^{150}Nd/^{144}Nd$. JNdi-1 was the most frequently measured standard during this study and its reproducibility (3.1 for $^{142}Nd/^{144}Nd$) is taken as representative of the external error for all samples."

Line 84. Three samples were measured during session 1 and were normalized to JNdi-1 after being corrected using $^{142}Nd/^{144}Nd_{AMES,session\ 1}$ / $^{142}Nd/^{144}Nd_{AMES,session\ 2}$.

Comment: Two points:

1. I don't know what is going on here, but it does not have a good appearance. All analyses need to be treated and normalized in exactly the same fashion. I suggest removing the data instead of making a special pleading argument here.

2. You explain how 3 samples were normalized to the standard analyses. How were the others normalized? Important to provide an explicit comparison here.

We agree that consistent data treatment is essential for scientific integrity. The distinct normalization approach for three of our samples was necessitated by a change in available reference materials between analytical sessions. All samples measured during Session 2 (the vast majority of our dataset) were directly calculated in the mu notation using JNdi-1, as generally accepted in the literature. The three samples from session 1 were analysed along with the AMES Rennes std, before JNdi-1 was available in our laboratory. To ensure these data were comparable to the rest of the dataset, we applied an inter-session normalization using the AMES Rennes Nd standard, which was measured consistently in both sessions. This step normalized Session 1 data to Session 2 data.

Modified in the Supplementary material lines 94–104: "All other Nd isotope ratios are given in the mu notation following the equation $\mu^{x}Nd = [(^{x}Nd/^{144}Nd_{sample} / ^{x}Nd/^{144}Nd_{terrestrial\ reference}) - 1] \times 10^6$, where the terrestrial reference composition for this study is the widely used JNdi-1 pure Nd solution standard. All the samples analysed during Session 2 were measured concurrently with JNdi-1 and their mu values were calculated directly. However, three samples were measured during Session 1. To ensure proper comparison of these data with the rest of the dataset, their measured isotope ratios were first normalized to Session 2 using the AMES Rennes Nd measured during both sessions, following the equation $^{x}Nd/^{144}Nd_{corrected\ sample} = {}^{x}Nd/^{144}Nd_{measured\ sample} \times [^{x}Nd/^{144}Nd_{AMES,\ session\ 2} / ^{x}Nd/^{144}Nd_{AMES,\ session\ 1}]$. The mu values for these corrected samples were then calculated similarly to the rest of the dataset."

Line 58. About 800 ng of Nd per analysis was loaded onto zone refined 99.999% Re filaments used in a double filament configuration.

Comment: Folks at Carnegie have indicated that large loads can yield anomalously high 142Nd/144Nd. The mechanism for this is not clear—likely owing to fractionation on the filament--but the result is that Nd load sizes must be incredibly consistent from sample to sample (and consistent between samples and standards).

This is an interesting observation! As far as we are concerned, we use typically less material than other recent studies investigating ^{142}Nd isotope compositions in terrestrial samples (800 ng in this study vs. 1000 ng in Garçon et al., 2018, Chem. Geol. for instance). Moreover, we always load the same amount of Nd onto the filament, extremely consistently and with a minimum spread of the deposit (<2 mm). We intend to reach the best possible conditions to allow highly reproducible measurements.

Modified in the Supplementary Material lines 59–60: "For both standards and samples, about 800 ng of Nd per analysis was loaded onto zone-refined 99.999% Re filaments used in a double filament configuration".

Line 102. We monitor these Nd ratios for variations exceeding the JNdi-1 reproducibility, indicating possible and associated ppm-level μ^{142}Nd deviations. DR1802-1 has ^{148}Nd and ^{150}Nd excesses (15.6 and 40.4 ppm) and a ^{145}Nd deficit (2.2 ppm – Table S1), mimicking NFS-induced variations (in red in Fig. S2B and Table S1). This suggests that its measured positive μ^{142}Nd might be underestimated and should be even more positive.

Comment: It's important for the reader to evaluate this. Please provide plots of µ142Nd vs µ145Nd, µ142Nd vs 148Nd, µ142Nd vs µ150Nd. Plot three different data groups in the plots: JNdi analyzed during the study, all Fani Maoré analyzed during the study, and all Mayotte analyzed during the study. Please show 2SE error bars on all the analyses. This will help evaluate fractionation influences are at work.

These plots have been added to the Supplementary Material as Figure S3.

Line 58. High-precision Nd isotope analyses were performed on a Nu Instruments TIMS at IPGP following a 5-line acquisition method8.

Comment: Please provide a visual in the paper that shows the cup used for each step for the 5 steps.

The cup configuration, as well as the method we use in this study are thoroughly detailed in Israel et al., 2025, Chem. Geol. and we do not feel it necessary to add it.

We now refer to the visual of the cup configuration by adding reference to Israel et al., 2025.

Modified in the Supplementary Material lines 57–59: "High-precision Nd isotope analyses were performed on a Nu Instruments TIMS at IPGP following a 5-line acquisition method thoroughly detailed in Israel et al., 2025[8] (cup configuration in their Figure 1)."

Line 211. Incorporation of <0.5% of such recycled sediment (GLOSS-II) is sufficient for its low ε^{143}Nd to balance the high ε^{143}Nd of Hadean bridgmanite and reproduce the value measured for Fani Maoré lavas, while not changing the proportions of Hadean bridgmanite and modern mantle components.

Comment: An issue here is that the GLOSSII ^{143}Nd/^{144}Nd ratio is not known.

We apologise if our explanations were confusing. We have revised the text to clarify our modelling approach, added values in Tables S2 and S3 and added a schematic (Fig. S6) illustrating the model calculations. We hope that it is now clear.

Modified in the Supplementary Material lines 274–278: "Here we use GLOSS-II (Global Subducted Sediments II), the average composition of subducted sediments suggested by Plank[38] and assume that the recycled sedimentary material has an age of 2 Ga, which implies no m^{142}Nd anomaly. We calculate its present-day Nd isotopic evolution (Table S3) using its published ^{143}Nd/^{144}Nd and Sm/Nd ratios[38] (Table S2) and the recycling model calculations of Chauvel et al. [35] as illustrated in Figure S6."

Referee #2:

Review of C. Israel et al., for Nature
"Hadean bridgmanite in the source of a present-day ocean island"

This study reports positive 142Nd anomalies (3.6 to 5.5 parts-per-million (ppm) above the Nd standard) in several volcanic rock samples from the Comoros Archipelago. Because such 142Nd anomalies cannot form after the extinction of 146Sm (half-life: 92 Ma), their presence indicates the incorporation of silicate differentiation products in the mantle source of the Comoros Archipelago formed within the first 500 Ma of Solar System history.

The early history of Earth is one of the most fascinating—yet least constrained—topics that isotope geochemistry can help illuminate. The manuscript's introduction is well written and clearly presents both the hypothesis and the motivation for this study. The paper also reports a substantial dataset from challenging and technically demanding analyses. However, I do not see evidence that the analytical methods represent an improvement over previous studies (e.g., Hyung et al., 2020; Horan et al., 2018; Peters et al., 2018), and I find that the authors' conclusion is not necessarily justified by the data presented.

Looking at the supplementary Table SD1A, the samples that were identified having 142Nd anomalies are reported to have 2SE errors of 1–2 ppm, allowing them to be distinguished from the JNdi-1 standard. However, these samples were measured only once. Other samples where repeated measurements were performed have 2SD errors that are on average ~4.2 ppm. This suggests that the 2SE errors of 1–2 ppm of samples measured once are underestimated, and that the 142Nd anomalies would not be significantly different from the terrestrial standard's long-term reproducibility of ±3 ppm. It is therefore unclear how this study overcomes the analytical limitations noted in earlier work. The reported two-year long-term reproducibility of 3.1 ppb for the 142Nd/144Nd ratio in the reference material appears similar to previous published studies.

Even if there was 142Nd variability in the samples studied (but the 1–2 ppm resolution would have to be tested by repeated measurements of samples), the data do not seem to require the authors' preferred interpretation—that remnants of a bridgmanite reservoir exist. Alternative explanations, such as recycling of early Hadean crust into the mantle (e.g., Tusch et al., 2022), could be equally plausible. Consequently, I am not convinced that this paper represents a significant advance in understanding that will influence thinking in the field, and would consider publication in another less-selective journal that allows for a longer format.

We would like to thank the reviewer for providing useful insights on our work. We understand the concerns raised here and have addressed them in order to demonstrate the robustness of our data and the importance of this study for the scientific community.

Precision, reproducibility and accuracy of the $^{142}Nd/^{144}Nd$ ratios of natural samples.

We agree that the distinction between internal precision (2 se) and long-term reproducibility (2 sd) is critical. As the reviewer mentioned, the 2 se values on single measurements differ from our long-term external reproducibility. While the 2 se values reflect the internal precision of single measurements, the long-term external reproducibility is given as 2 sd of our repeatedly measured pure Nd standards and natural rock reference materials as published in Israel et al., Chem. Geol, 2025. These standards and rock reference materials were run at the same period as Fani Maoré samples. Given the large sample quantity required per run (800 ng) and the extended duration of the analysis (18 hours to improve counting statistics), we chose to perform

multiple measurements on a subset of seven samples, out of 21, to demonstrate that their reproducibility is consistent with our long-term standard data. We thus consider that the 2 sd error on pure Nd standards is the best long-term estimate of reproducibility of the measured isotopic ratios during the analytical session and this is the most important error used to identify natural variations.

We have updated the supplementary material to include a sentence about the rock reference materials measured during the same analytical sessions to further support the robustness of our data.

Modified in the Supplementary Material lines 90–91: "Rock reference materials were measured during the same analytical sessions as our samples. Results were extensively described in Israel et al.[8] and show similar reproducibility."

Significance of the ^{142}Nd anomalies in Fani Maoré lavas.

The significance of the ^{142}Nd anomalies measured in natural samples is assessed not only by the reproducibility of the data but also by the distribution of the entire dataset for Fani Maoré laves.

- Six out of 13 samples are beyond the 3.1 ppm 2-standard deviation on JNdi-1.
- Three out of 13 Fani Maoré samples were measured three to four times. The majority of them are significantly higher than the μ^{142}Nd = 0 ±3.1 ppm value of JNdi-1.
- Among the 20 measurements we performed on Fani Maoré samples, 12 have anomalies beyond +3.1 ppm and only one measurement shows a negative anomaly. Even when considering the scatter of the data, the detection of 19 positive anomalies out of 20 is unlikely to be a coincidence.
- Considering the µ142Nd values of the 13 Fani Maoré samples to be a sufficient statistical dataset, their mean value of +3.2 ±0.9 (2 se, n=13) is beyond the mean value of the terrestrial reference (0 ±0.7 2 se, n=23).

We therefore think that the data are robust and that the lavas do show a positive anomaly. Unfortunately, we do not really see what could be changed in the manuscript or the supplementary material to convince the reviewer of the existence of the positive anomaly.

Alternative explanations for positive ^{142}Nd anomalies in present day volcanism.

We agree that alternative explanations, such as the recycling of early Hadean crust, should be considered and we modified the entire section of the main manuscript entitled "Origin of positive ^{142}Nd anomalies" to evaluate the potential role of a Hadean depleted reservoir. However, we do not think that recycling of an early Hadean crust is a viable process to generate a positive ^{142}Nd anomaly. The main reasons are:

- A Hadean protocrust such as that proposed by Turner et al., 2025, Nature, or a more classical continental crust, is LREE-enriched and has a lower-than-BSE Sm/Nd ratio. Such material would produce a negative rather than a positive ^{142}Nd anomaly during the short lifespan of ^{146}Sm.
- For such material to produce a positive ^{142}Nd anomaly, the crust would need (a) to originate from an already depleted mantle with a positive μ^{142}Nd value and (b) to form late in the Hadean, after the majority of the ^{146}Sm had decayed and could no longer affect the ^{142}Nd/^{144}Nd ratio. While this scenario is not impossible, it is highly improbable.
- An early felsic material such as Isua noritic dykes has been shown to have a positive μ^{142}Nd ($\leq$ +20), but due to its low Sm/Nd ratios, it also has very negative ε^{143}Nd values (≈-30 – see Rizo et al., 2012, Nature). Mass balance calculations demonstrate that the positive μ^{142}Nd and ε^{143}Nd of Fani Maoré cannot be reproduced by incorporating such material.

By contrast, the crystallisation of the magma ocean was the first major event capable of generating large-scale mantle heterogeneities, producing both depleted and enriched domains. In our view, preservation of a remnant of such depleted material in the deep mantle provides a more robust and geochemically consistent explanation for the positive ^{142}Nd anomaly found in Fani Maoré than subduction and the long-term survival of a small volume of crust.

Line-by-line comments:

• Line 42: heterogeneities of the Hf-W and I-Xe short-lived chronometer can be explained by events after the 50-100 Ma of the Earth (e.g., recycling Hadean crust into the mantle or core-mantle interactions) and thus should be rephrased for clarity.

Modified in the main manuscript lines 41–44: "In addition, isotopic heterogeneities of the Hf-W and I-Xe short-lived geochronometers have been reported in OIB, thereby testifying to metal-silicate differentiation events and heterogeneous accretion of volatile-rich materials during the first 50 to 100 Ma of the Earth history[5–7]."

• Line 50: Many Archean samples display 142Nd variability, but not many locations with those Archean samples. Text should be rephrased for clarity.

Modified in the main manuscript lines 51–53: "Some Archean samples preserved in continental settings display excesses and deficits in ^{142}Nd up to 20 parts per million or ppm (see compilation in [10]) testifying of the active decay of ^{146}Sm"

• Line 223: Correct the citation.

Modified in the main manuscript lines 232–233 "Chiera, N. M. *et al.* The 146Sm half-life re-measured: consolidating the chronometer for events in the early Solar System. *Sci. Rep.* **14**, 17436 (2024).".

• Supplementary material: In the first analytical session where the Nd isotope standard AMES #13–#17 was measured, several values in Table SD1B appear anomalous. Some multidynamic and dynamic ratios—particularly the stable 145Nd/144Nd ratio—are off. Also, the 142Nd/144Nd "Static 1" ratios are also off (1.1417 instead of ~1.1418). If any samples were measured during this session and normalized to these standards, the authors should re-examine those results.

The multi-dynamic and dynamic ratios of AMES #13-17 were measured at the end of analytical session 1, before changing the Faraday cups and as noticed by the reviewer some of the static measurements started to deviate significantly. These differences testify here of the importance of changing the Faraday cups to obtain more accurate data, especially as they significantly impact the static ratios. This has been discussed at length in the analytical paper that we published earlier this year (Israel et al., 2025, Chem. Geol.). We agree that the static 1 ratio for ^{142}Nd/^{144}Nd is extremely low for AMES #13-17 but it is not the case of the multi-dynamic average value. As explained in the text, the supplementary material and in literature (e.g., Garçon et al., 2018, Chem. Geol. for Nd and Di et al., 2021, Chem. Geol. for Sr), multi-dynamic measurements are much more robust and reproducible than static measurements. Here, as mentioned on line 57 of the Supplementary Material, all results used in the discussion are multi-dynamic measurements.

Only three measurements on natural samples were performed during session 1, along with AMES #13-17, including only one samples from Fani Maoré. If we do not include that sample, the Fani Maoré average value becomes μ^{142}Nd = +3.4 ±3.1/±0.9 (2 sd/2 se, n=12). This does not change the average value and our interpretation.

Manuscript Number 2025-06-16053

We would like to thank the editor and the two reviewers for their constructive comments. We would also like to thank the editor for giving us the opportunity to appeal their decision. We were indeed surprised that the manuscript was not given the opportunity to be re-revised, since the reviewers' comments were largely positive (one even suggested publication) and their concerns easily addressable. We took all comments into account, and we believe that the manuscript addresses all of the reviewer's concerns.

Please see our responses to all comments below, shown in blue. Changes made to the manuscript itself are indicated in light blue, and a version of the manuscript with highlighted changes is also provided. We hope that the answers to the various comments and the changes made to the manuscript are suitable.

Referee #1:

The authors have done a really good job convincing me that there is a 142Nd anomaly in their data. The time series 142Nd plots and the plots showing the XXXNd vs 142Nd plots show that there is excess 142Nd in their rocks compared to the standard. This does not appear to be caused by fractionation.

We would like to thank the reviewer for this extremely positive feedback!

However, there are two remaining items that need to be addressed, one easy and the other more difficult:

1. To ensure there is no contribution from 142Ce on the 142Nd, the authors should include a figure of 140Ce/144Nd vs 142Nd/144Nd and put this plot in the supplement. This should be an easy 10-minute job.

This is a perfectly understandable request that helps visualizing the absence of impact of the Ce isobaric interferences on our data.

We added a **new Figure S3** in the Supplementary material showing the absence of relationship between monitored Sm and Ce interferences and $^{142}Nd/^{144}Nd$ ratios for Fani Maoré and Mayotte samples.

We also added a short description on lines 112-118 in the Supplementary Material: "Samarium and Ce interferences were essentially inexistant for most samples. Nevertheless, six measurements were corrected for a ≈0.5 ppm Sm contribution and DR1402 for a 2.0 ppm contribution on $^{142}Nd/^{144}Nd$ (Supplementary Data 1A). Similarly, Ce interference was negligible during most measurements, with only three requiring corrections, with contributions of 1.6 ppm (DR140401) and 6-8 ppm (both measurements of DR070202) on $^{142}Nd/^{144}Nd$. Interference-corrected analyses are unrelated with the extreme $^{142}Nd/^{144}Nd$ ratios reported in this study (Fig. S3)."

2. The issue of the origin of the 142Nd excess in their lavas is a bit more complicated. The authors argue that the 142Nd excess is due to bridgmanite, but reviewer #2 and I both agree that this is not a unique explanation for the excess 142Nd. This is the only remaining issue that lingers. I appreciate that the authors have augmented their discussion to address this issue.

We acknowledge the reviewers' valid point: our privileged interpretation is not the only plausible one, and we did not adequately assess the alternative model. We have revised our discussion to address this oversight. In the updated version, we restructured the paper to present both possible origins (see section titles). We now present and quantify the two competing models for the origin of the Fani Maoré basalts:

1) Shallow origin (crustal extraction model): This scenario requires between 28% and 90% of the source material to have formed over 4.3 billion years ago.
2) Deep origin (bridgmanite crystallization model): This scenario requires only 9 to 11% of the source material from the lower mantle.

This raises a critical question: Which scenario is more scientifically plausible?

1) Preserving nearly the entirety of a small, ancient piece of mantle (which underwent crystal extraction 4.3 billion years ago) throughout geological time in a convecting mantle?
2) Or tapping into just 10% of the lower mantle's major mineral constituent?

We argue that the deep mantle origin is the more quantitatively reasonable explanation. However, we now present both models more clearly, enabling readers to evaluate them and choose the one they find most compelling.

Without the new experimental data and the bridgmanite ^{142}Nd model, our paper would have been limited to the shallow origin—an interpretation many colleagues would rightly consider highly unlikely. By presenting both possibilities, we provide a more comprehensive framework for understanding the formation of these basalts. While we advocate for the deep origin, we respectfully leave the choice open to the reader.

A. The authors write: "mantle depletion is only firmly recorded [in zircons] after ~3.8 Ga". Comment: The zircons record enriched mantle reservoirs prior to 3.8 Ga. Mass balance requires there were complementary depleted reservoirs. I'm not sure how the zircon record excludes the presence of depleted reservoirs prior to 3.8 Ga.

The section mentioned by the reviewer (lines 85–86 in old manuscript) has been entirely modified. We now start the discussion by evaluating a potential origin in an early mantle depleted by continental crust formation (see lines 80-94 of the main manuscript).

The model is also discussed in the supplementary material (**new section** lines 306–341 "**What about a Hadean depleted mantle formed through continental crust extraction**?") and the quantitative results are shown in the **new Figure S9**. We estimate that with a crustal extraction age of 4.3 Ga, the source of Fani Maoré would consist of almost 90% of this Hadean material, a quantity that appears difficult to preserve since the Hadean.

The specific sentence mentioned by the reviewer has been removed from the text and a modified version is now present in the Supplementary Material (see lines 309–313): "These anomalies have been attributed either to early crustal extraction producing a depleted mantle residue[35,36,38] or to large-scale processes generating heterogeneities[39–41]. However, there is no clear evidence for large volumes of Hadean crust[42,43], and only rare evidence of mantle depletion before ~3.8 Ga[44]; most crustal growth models also favour progressive rather than massive early extraction[45]."

B. The authors write: "While the survival of localised Hadean depleted domains cannot be ruled out, the constraints provided independently by geophysical and geodynamical studies focusing on mixing time in the mantle, make this option a low-probability scenario." Comment: I'm not sure the studies cited provide probabilities of one scenario occurring vs the other scenario occurring. The probability assigned is the authors' perspective, but not really evaluated in the studies cited.

As mentioned above, this section has been completely rewritten, and the sentence no longer exists. Instead, we now summarize the conditions necessary for an early depleted mantle formed through crustal extraction to account for the positive Nd anomaly measured in Fani Maoré (see lines 88–94 of the main manuscript).

All details are also provided in the Supplementary Material in the section entitled “**What about a Hadean depleted mantle formed through continental crust extraction?**” (lines 306–341 of the Supplementary Material).

C. To frame this a bit differently, the authors would need to exclude the presence of Hadean depleted reservoirs before they can convince the community that bridgmanite is the only remaining viable explanation for the ^{142}Nd in their lavas. I don’t think this can be done with existing evidence, but I would be very happy if the authors could find such evidence. Alternatively, the authors can simply acknowledge that bridgmanite is simply one possible explanation, but survival of early depleted mantle domains cannot be excluded. Upper mantle material is sent to the lower mantle today (depleted mantle lithosphere attached to slabs, etc), and upper mantle communication with lower mantle was not unlikely in the Hadean. Upper mantle sent into the lower mantle can be preserved for long time periods due to slower convective motions in the lower mantle. I have no skin in this game—I would be perfectly happy to be convinced that bridgmanite is the only explanation here—but I am not yet convinced that bridgmanite is the only explanation. The authors either need to acknowledge their (very interesting) data do not have a unique explanation in this regard, or they need to convince me and the other reviewer that bridgmanite is the only explanation. In either case, I would be supportive of publication.
In this revised version of the manuscript, we present the scenario of a Hadean-depleted mantle formed by crustal extraction more clearly. We also evaluate the likelihood of reproducing the μ^{142}Nd isotope composition of the Fani Maoré samples by incorporating this material into its source. This is clearly stated in the main manuscript (lines 80–94) and further elaborated on in the Supplementary Material (lines 306–341).

We do indeed favour a magma-ocean-crystallisation scenario because it is able to produce much more positive isotope signatures, implying less material (~10%) in the source of Fani Maoré (the crustal extraction model requires up to 90%, which is enormous and quite frankly, hard to imagine), and a greater chance to survive in the mantle over the Earth’s history. Furthermore, if the formation of a Hadean continental crust is still a matter of debate, the existence of the magma ocean is widely acknowledged in the community and its crystallisation is undisputable.

Referee #2:

The revised manuscript is well written, and the newly added discussion on early crust extraction, as well as the updates to the supplementary materials, represent meaningful improvements. I also acknowledge the substantial amount of work that has gone into this study. However, as Carl Sagan famously remarked, "extraordinary claims require extraordinary evidence," and the data presented here are not as unequivocal as the authors suggest.

We would like to thank the reviewer for the acknowledgment over the quality of our work and the improved discussion.

Regarding the significance of the reported 142Nd anomalies in Fani Maoré lavas: in their response, the authors state that "unfortunately, we do not really see what could be changed in the manuscript or the supplementary material to convince the reviewer of the existence of the positive anomalies." However, it is not only me who needs to be convinced—any claim of statistically significant 142Nd deviations must be rigorously supported for the broader community. As currently written, the manuscript makes statements (e.g., "the significant 142Nd excess in Fani Maoré lavas…" in line 73, or line 190) that require quantitative justification.

If the co-authors wish to assert that these anomalies are significant, this must be demonstrated using appropriate statistical approaches. For example, a test comparing the sample values with the long-term JNdi-1 average (given the reported reproducibility of 3.1 ppm) could be used to show that the probability of the observed offsets occurring by chance is <5% (i.e., p<0.05). Without such analysis, these statements are not yet supported.

We thank the reviewer for the suggestion. We should have done it right from the start! We now provide a statistical evaluation of the distinction between the standard and the Fani Maoré samples. A sentence was added in the main text lines 71–73 and a paragraph was added in the Supplementary Material:

- we first show the absence of overlap when using the error bars.
  This can be found in the Supplementary Material lines 145–146 "The 2-standard errors on the mean value for Fani Maoré and JNdi-1 do not overlap as +3.2 ±0.9 (Fani Maoré, n = 13) is distinct from 0 ±0.7 (JNdi-1, n = 23).
- we calculate a t-test and show that the p-value is 8.9×10^{-6} which is drastically below the 0.05 threshold to conclude that both populations are statistically distinct.
  This can be found in the main text lines 70–72 "This positive μ^{142}Nd value is significantly different from the JNd-1 value as statistically demonstrated by a p-value of 9×10^{-6} much below the threshold at 0.05 (see Supplementary Material for further details)." and in the Supplementary Material lines 146–150 "The distinction between Fani Maoré and JNdi-1 populations is also assessed with a t-test providing a p-value of 8.9×10^{-6}, where two populations are considered as statistically distinct whenever the p-value is below 0.05. On the opposite, the p-value when comparing Mayotte and JNdi-1 is 0.14, thus showing that they are statistically undistinguishable.")
- we added a **new Figure S6** showing the distribution of the data with histograms and gaussian distribution curves.
  The text was also modified in the Supplementary Material lines 150–153 "The distribution Fani Maoré samples in histograms also points toward a strict distinction from JNdi-1 (Fig. S6) as the maximum of their gaussian distribution curve is distant by 3.5 ppm from JNdi-1, with 95% of Fani Maoré data located between +2 and +5 while 95% of JNdi-1 measurements are located between -2 and +2"

Also, line 63 states "This enhanced precision overcomes the analytical limitations of previous studies…". As noted in my previous review, the precision reported here does not

exceed that of several published works. For example, both Saji et al. (2016) and Wang and Carlson (2022) reported ~ 2.5 ppm precision for 142Nd/144Nd ratios on the JNdi-1. Therefore, previous publications seem to report a higher precision compared to this study. Line 63 should be revised to accurately describe the analytical capabilities of this study.

We apologize for unclear terms in our description of the precision. We agree that short-term reproducibility reported by Saji et al. (2016) and Wang and Carlson (2022) are similar to our short-term reproducibility. However, to our knowledge, our study is the first one that reports a such reproducibility over a several-year-long period of time.

The text is now modified in the Main Manuscript lines 60–66 "Using a new 5-line multi-dynamic method for Nd isotopes by TIMS[22], we achieve a 2-year long-term reproducibility of 3.1 ppm (2sd) on the $^{142}Nd/^{144}Nd$ ratio of the reference material. Over individual periods of a few months, our reproducibility is identical (see Supplementary material) and corresponds to the best published errors[23,24]. The reported long-term precision is the first of its kind and provides confidence for the identification of remnants of Hadean material in the modern mantle."

We also better describe our short-term and long-term reproducibility in the Supplementary material lines 82–87 "The short-term reproducibility, which is usually reported in literature as being equivalent to uninterrupted series of samples and standard measurements, is similar. We identify six series (March–May 2022; July 2022; December 2022–January 2023; April–June 2023; August–September 2023; November 2023–January 2024), the reproducibility of which on the $^{142}Nd/^{144}Nd$ ratio ranges from 1.8 to 4.1 ppm (n = 4–9, excluding n ≤ 3), giving an average reproducibility of 3.1 ppm."

Given these unresolved issues, I remain unconvinced that the present study meets the evidentiary threshold required for publication in Nature.

We hope that the addition of the statistical treatment and the clarification provided for the reported reproducibility of the data fulfil the requests made by the reviewer.

Manuscript Number 2025-06-16053:

We would like to thank the editor and the two reviewers for their constructive comments throughout this entire process. We took all the final comments into account, and we believe that our manuscript now fully addresses all of the reviewer's concerns.
Please see our responses to all comments below, shown in blue. Changes made to the manuscript itself are indicated in light blue, and a version of the manuscript with highlighted changes is also provided. We hope that the answers to the various comments and the changes made to the manuscript are suitable.

Referee #1:

Summary:

The authors have done a thorough job or reframing the arguments in the manuscript to better reflect uncertainties in how 142Nd anomalies are preserved in the modern convecting mantle. I actually enjoyed reading the revised arguments, particularly the new argument acknowledging that the 142Nd anomaly can be generated in the upper mantle--and such an anomaly could explain the anomalous 142Nd in OIB--but they use a strong geodynamic argument to explain why an upper mantle origin is unlikely.

Additionally, I would like to point out that they have convincingly shown that they can resolve small 142Nd anomalies in OIB. Their long-term precision is really extraordinary, but they have made a series of diagrams (Figs S2, S3, and S4) that go a long way toward convincing me that the 142Nd anomalies are real. The instrument (Nu TIMS) uses fully modern electronics (the electronics in the Thermo Tritons are >30 years old!) and I believe this allows them to push the frontiers of high-precision analysis.
We thank the reviewer for this very positive feedback!

Below I provide very minor comments that request a bit more clarity in the authors' language. Otherwise, this manuscript is ready for prime time. I hope that it can be published without delay.

Specific comments:

1. The authors wrote: The survival of localised Hadean depleted domains originating from the upper mantle thus cannot be ruled out but meeting the conditions for it to have survived up to today in significant amounts makes this option a low probability scenario.

Comment: As requested in my prior review, the authors go through the exercise of showing that depleted upper mantle domains can generate the 142Nd anomalies that they observe in OIB. Critically, they go one step further and show, using geodynamic arguments, that it is difficult to preserve a 142Nd anomaly in an upper mantle reservoir, making its preservation a low probability event. This is an ideal response, and makes their preferred interpretation--142Nd anomalies are generated by bridgmanite formation from a magma ocean--a more realistic model.

Here again, we thank the reviewer for this very positive feedback!

2. Sizes of reservoirs. The following 4 comments are related.

A. The authors wrote: To match the µ142Nd of +3.2, and the e143Nd measured at Fani Maor., requires ~90% of this material in the source (see Supplementary Material for details), a volume

so significant that it raises serious questions about its preservation30 within the convecting mantle for ~4.3 billion years.
Comment: I'm not sure I follow what "material in the source" refers to. Are the authors suggesting that 90% of the entire mantle is bridgmanite, or 90% of the plume? If 90% of the mantle is bridgmanite with the µ142Nd of +6, then it's easy to preserve the +6 anomaly for 142Nd--most of the mantle is made of this material--so no serious questions are raised.
Thanks for highlighting this ambiguity. We modified the text lines 87-90 and hope that it is now clearer: "Matching the Fani Maoré μ^{142}Nd and e^{143}Nd values requires the plume to consist of ~90% of this depleted material (see Methods for details), a volume so significant that it raises serious questions about its preservation[30] within the convecting mantle for ~4.3 billion years."

B. The authors wrote: Mitigating this requires identifying a process that generates a reservoir which is (1) large enough for a small fraction of it to realistically be preserved,
Comment: Again, I'm not sure I follow. The authors seem to suggest that it is difficult to find a process that preserves a small part of a very large reservoir with positive 142Nd. I'm not sure I see the problem here. Larger reservoirs are easy to preserve than smaller reservoirs.
Thanks again for highlighting this ambiguity. We modified the text lines 90-93 and hope that it is now clearer: "Ideally, we need a process that generates a reservoir which is either large enough so that a small fraction of it can realistically be preserved; and/or a reservoir that has a much more positive ^{142}Nd anomaly so that smaller quantities would produce the observed effects."

C. The authors wrote: has a much stronger positive 142Nd anomaly so as to necessitate minute quantities to produce the observed effects.
Comment: Why does the reservoir with positive 142Nd have to be smaller (in mass) and have larger 142Nd? Why is it necessary (the authors write necessitate) that the positive 142Nd reservoir is sampled in minute quantities?
Thanks for the comment about the lack of clarity. This comment relates to the sentence mentioned in comment B. We hope that the revised text clarifies the message.

D. The authors wrote: For a depletion age of 4.46 Ga, the depleted reservoir would have a µ142Nd of +19 and for a depletion age of 4.3 Ga it would be +6 (Fig. S9). Using these values and the Nd concentration of DMM46, we calculate that about 26% of such material is required to reproduce Fani Maor. µ142Nd if the depletion occurred at 4.46 Ga. Any later formation age for the depleted reservoir involves higher proportions in the source of Fani Maor., up to ≈70% for an age of 4.3 Ga.
Comment: The amount of material required to reproduce Fani Maore depends on the µ142Nd (µ142Nd = +19 vs µ142Nd = +6). Each unique µ142Nd will require a unique percentage of material. Provide both in a clear way.
We understand that the sentence was confusing. This sentence was in the supplementary material, and we have now modified it so that the proportion of required depleted material is given for both ages (4.46 and 4.3 Ga). See lines 692-696: "For a depletion age of 4.46 Ga, the depleted reservoir has a μ^{142}Nd of +19 and for a depletion age of 4.3 Ga it has a μ^{142}Nd of +6 (Extended Data Fig. 9). Using these values and the Nd concentration of DMM[27], we can calculate how much material is required to match the Fani Maoré μ^{142}Nd value. If the material

is 4.46 Ga-old, it must represent 26% of the plume source, and if it is 4.3 Ga-old, it must represent 70% of the plume source."

E. In that case, the proportion of Hadean bridgmanite would be much higher (>30 %), making it difficult to envisage how such large quantities could be present in the mantle today. Comment: This seems to be a different version of earlier arguments about reservoir size. In this case, the argument is that the authors question how a relatively large reservoir could be preserved over 4.5 Gyr. The issue is that this is framed as an opinion, without supporting evidence. If it is difficult to preserve this mass of Bridgmanite for 4.5Ga, explain why and provide citations. This will make the argument stronger.
Thanks for the comment. We realize that this sentence was not necessary and was only bringing confusion. We remove it (see line 186); by doing this we shorten slightly the text as requested by the editor.

F. The shallow model requires the source to contain 28–90% of Hadean material, whereas the deep model only requires 9–11%.
Comment: See previous comment ("comment E"). Whether a reservoir or one size (28-90%) or another (9-11%) can be preserved is framed as an opinion, so this is not a strong argument. In the supplement, the authors use a logical and convincing geodynamic argument to deal preservation of the former (upper mantle) reservoir, where they write: "For Fani Maore's source to be largely composed of such depleted mantle requires that the Hadean component is large enough to be preserved unaltered for more than 4 Gyr. As it formed in the upper mantle, it needs to be dense enough to sink at the bottom of the lower mantle where it could potentially escape the efficient stirring due to convection." I strongly suggest that the authors use the same argument here."
Thanks for the comment and for highlighting that this sentence was only presenting an opinion and was not necessary. We prefer to delete it instead of adding an extra couple of lines by moving the text present in the supplementary material to the main text. By doing so, here again, we shorten slightly the text as requested by the editor. The text now reads (see lines 184-188): "The shallow model requires the source to contain 28–90% of Hadean material, whereas the deep model only requires 9–11%. Independently, recent geodynamical fluid dynamics simulations of magma ocean crystallisation[35] show the widespread production of geochemical (Sm/Nd and Lu/Hf) heterogeneities during solidification and their vertical scattering throughout the mantle.".
